# Mitogen Activated Protein Kinases in Steatotic and Non-Steatotic Livers Submitted to Ischemia-Reperfusion

**DOI:** 10.3390/ijms20071785

**Published:** 2019-04-10

**Authors:** Mónica B. Jiménez-Castro, María Eugenia Cornide-Petronio, Jordi Gracia-Sancho, Araní Casillas-Ramírez, Carmen Peralta

**Affiliations:** 1 Transplant Biomedicals S.L., 08042 Barcelona, Spain; monicabjimenez@hotmail.com; 2 Institut d’Investigacions Biomèdiques August Pi I Sunyer (IDIBAPS), 08036 Barcelona, Spain; cornide@clinic.ub.es; 3 Liver Vascular Biology Research Group, Barcelona Hepatic Hemodynamic Laboratory IDIBAPS, 08036 Barcelona, Spain; jgracia@clinic.ub.es; 4 Centro de Investigación Biomédica en Red de Enfermedades Hepáticas y Digestivas (CIBERehd), 08036 Barcelona, Spain; 5 Hospital Regional de Alta Especialidad de Ciudad Vitoria, Ciudad Victoria 87087, Mexico; 6 Facultad de Medicina e ingeniería en Sistemas Computacionales de Matamoros, Universidad Autónoma de Tamaulipas, Matamoros 87300, Mexico

**Keywords:** ischemic-reperfusion injury, non-alcoholic fatty liver disease, mitogen activated protein kinases, steatosis

## Abstract

We analyzed the participation of mitogen-activated protein kinases (MAPKs), namely p38, JNK and ERK 1/2 in steatotic and non-steatotic livers undergoing ischemia-reperfusion (I-R), an unresolved problem in clinical practice. Hepatic steatosis is a major risk factor in liver surgery because these types of liver tolerate poorly to I-R injury. Also, a further increase in the prevalence of steatosis in liver surgery is to be expected. The possible therapies based on MAPK regulation aimed at reducing hepatic I-R injury will be discussed. Moreover, we reviewed the relevance of MAPK in ischemic preconditioning (PC) and evaluated whether MAPK regulators could mimic its benefits. Clinical studies indicated that this surgical strategy could be appropriate for liver surgery in both steatotic and non-steatotic livers undergoing I-R. The data presented herein suggest that further investigations are required to elucidate more extensively the mechanisms by which these kinases work in hepatic I-R. Also, further researchers based in the development of drugs that regulate MAPKs selectively are required before such approaches can be translated into clinical liver surgery.

## 1. Introduction: Ischemia-Reperfusion, MAPKs and NAFLD patients

The hypoxia and subsequent oxygen delivery restoration to the liver, namely, hepatic ischemia-reperfusion (I-R) induces deleterious effects in the surgery of resections and liver transplantation [1,2,3,4]. The shortage of organs has led centers to expand their criteria for the acceptance of marginal grafts [5] among which, steatotic livers are conspicuous [1]. In non-alcoholic fatty liver disease (NAFLD), the presence of fatty infiltration might be associated with inflammation resulting in non-alcoholic steatohepatitis (NASH) [6]. The prevalence of obesity in society increases the incidence of NASH, a crucial risk factor since steatotic livers tolerate poorly to I-R damage resulting in liver dysfunction and regenerative failure as well as primary non-function after surgery [7,8,9,10,11,12]. Investigations focused in evaluating the hepatosteatosis mechanisms might be useful in the establishment of specific therapies to prevent hepatic I-R injury [13,14]. 

It has been widely described that NAFLD implies the presence of fatty infiltration, inflammation, cell death and collagen deposition in the liver tissue. Mitogen-activated protein kinases (MAPKs) are mediators that participate in the signaling mechanisms underlying these cellular events and therefore, could be considered as possible therapeutic targets.

The three well-characterized subfamilies of MAPKs are p38, c-Jun N-terminal kinase (JNK) and extracellular signal-regulated protein kinases (ERK 1/2) and are 60–70% identical to each other. However, p38 and JNKs respond to stress stimuli whereas ERKs are phosphorylated by proliferative stimuli [15,16,17,18,19]. Depending on the pathological conditions that occur, MAPKs play different biological roles. In order to evaluate the usefulness of the MAPK as possible therapeutic targets, it is necessary to distinguish which are the functions that have been described for the MAPK in each of the stages that comprise the NAFLD and should also consider the influence of comorbidities. At the clinical level, there are currently very few studies that have evaluated the role of MAPK in patients with NAFLD, and such studies point to an inflammatory role of MAPKs in NASH with or without obesity, as exposed in the following.

It has been reported that vitamin D suppresses several pathways involved in inflammation and cytokine production including MAPK and nuclear factor-κ Beta (NF-kB) [20,21,22]. Recently, research work has demonstrated that vitamin D deficiency is associated with increased risk of NASH. In patients with NAFLD and vitamin D deficiency, an up-regulation of the MAPK and NF-kB inflammatory pathways was observed. Since increased accumulation of macrophages in adipose tissue is a hallmark of obesity and NAFLD, these studies provide a mechanistic link between vitamin D deficiency and the inflammatory response, which is a key pathogenic event in NAFLD [23]. Of interest, dietary vitamin D supplementation decreased MAPK and NF-kB activation in a murine model of colon cancer [23]. 

An exploratory study examined patterns of MAPK expression in obese patients. In NASH patients, activation of p38 MAPK was detected in a hepatic biopsy. It is well known that p38 MAPK participates in the regulation of inflammatory response [24]. The involvement of the apoptosis signal-regulating kinase 1 (ASK1)-MAPK pathway, the transducer activator transcription 3 and other survival pathways insulin receptor substrate 2 via phosphoinositide 3-kinase (PI3k) and its downstream effectors 3-phosphoinositide dependent protein kinase-1, ribosomal protein S6 kinase polypeptide 1 and v-protein kinase B (Akt) oncogene homolog) in NASH and NASH-related fibrosis has been also suggested [25,26]. 

Recently, elevated levels of advanced glycation endproduct (AGE) has been observed in the serum of NAFLD patients. Furthermore, accumulating evidence suggests that the binding of AGEs with their receptor (RAGE) results in the generation of reactive oxygen species (ROS) from activated nicotinamide adenine dinucleotide phosphate (NADPH) oxidase. This culminates in the activation of p38 MAPK and the translocation of NF-kB p65 into nucleus, leading to the transcription of growth factors, inflammatory cytokines, chemokines and adhesion molecules [27]. 

The pattern of MAPK expression in NAFLD patients submitted to surgical conditions requiring hepatic I-R has not been previously reported. As will be discussed below, at an experimental level, there exist results characterizing the role of MAPK in NAFLD, but not all studies distinguish which stage of NAFLD is paralleling the experimental model (simple steatosis, NASH, etc.). It is notorious that in different experimental models of NAFLD, MAPKs play a predominantly inflammatory role, as it seems the case at the clinical level. This supports a crucial role for MAPK in the underlying pathogenic mechanisms of NAFLD. Nevertheless, preclinical studies evaluating the role of MAPKs on fatty infiltration, damage and regenerative failure in surgical conditions of hepatic resections under vascular occlusion and liver transplantation in each of the stages that comprise the NAFLD have not been reported and intensive preclinical studies will be required to address this issue. 

## 2. Role of MAPKs in Experimental Models of Hepatic I-R Injury Using Steatotic and Non-Steatotic Livers

A large number of factors and mediators play a part in hepatic I-R injury [1,28,29,30,31]. The relationships between the signaling pathways involved are highly complex and it is not yet possible to describe, with absolute certainty, the events that occur between the beginning of reperfusion and the final outcome of either non-function or dysfunction after surgery. In addition, the mechanisms responsible for hepatic I-R injury depend on the type of the liver, which has made it so difficult finding effective therapeutic strategies to protect both liver types against I-R injury [1,32]. Among MAPKs, p38, JNK and ERK 1/2 are activated by a variety of cellular stresses, such as I-R [15,16,18,19,33,34,35,36,37,38,39,40] and are considered as a potential target in hepatic I-R [14,41]. 

As explained below, the role of MAPKs in steatotic livers undergoing hepatic I-R injury have been mainly focused on normothermic conditions. Indeed, in our view, there is only a paper evaluating the role of ERK in steatotic liver transplantation whereas the relevance of p38 and JNK remains to be elucidated. 

### 2.1. p38 in Hepatic I-R and Their Therapeutic Implications

#### 2.1.1. p38 in Hepatic I-R 

These proteins are activated by stress stimuli as those occurring in ischemic conditions, which would favor the inflammatory response [42,43]. Indeed, activation of the p38 pathway and p38-associated inflammatory processes play a crucial role in post-ischemic damage in steatotic and non-steatotic livers [35,40,44,45,46]. p38 is phosphorylated and activated after reperfusion [15,33,39]. Their activation is associated with the induction of apoptosis and necrosis [33,47]. In steatotic livers, p38 activation increased adiponectin levels thus inducing ROS generation and hepatic injury [48]. However, p38 inhibition after the pharmacological treatment with a p38 inhibitor, SB203580, reduced hepatic I-R injury in both type of livers [40,44]. These data indicate that p38 induces ROS generation, a key mechanism responsible for the vulnerability of steatotic livers to I-R. Further studies will be required to elucidate the potential involvement of antioxidants and ROS generating systems in the effects of p38 on ROS. In fact, previous studies from our group indicated less glutathione (GSH) and superoxide dismutase (SOD) levels and more alterations in antioxidants and ROS generating system (mitochondria and xanthine/xanthione oxidase) in steatotic livers than in non-steatotic livers as a consequence of hepatic I-R [2,49]. The benefits of the p38 inhibitor, SB203580, on cell death in both liver types could be of clinical interest since it could reduce necrosis and apoptosis in steatotic and non-steatotic livers, respectively. It has been suggested that necrosis rather than apoptosis is the predominant process by which steatotic livers undergo cell death [49,50,51]. Results based on an experimental model of total hepatic normotermic ischemia of 15 min indicated that hypoxia-reoxygenation increased p-p38 and p-JNK, but decreased p-ERK expression in steatotic hepatocytes. In contrast, pretreatment with astaxanthin regulated such MAPKs, thus protecting steatotic hepatocytes against the deleterious effects induced by hypoxia-reoxygenation, including apoptosis [52]. The relevance of the changes in the MAPK signaling pathway induced by astaxanthin on cell death remain to be elucidated. 

In our view the role of MAPKs in the different types of cells involved in the pathophysiology of hepatic I-R requires intensive investigation since the effects derived from the pharmacological modulation of MAPKs might be specific for each cell type. Consequently, this would result in the protection or harmful effects on hepatic I-R injury. Indeed, the effect of ASK1 is associated to opposite responses of steatotic hepatocyte and of steatotic Kupffer cells (KC) to hypoxia/reoxygenation damage. A lipid-stimulated ASK1 activation promotes the increased susceptibility to hypoxia/reoxygenation damage of steatotic hepatocyte through activation the cytotoxic axis ASK1/JNK. Accordingly, this indicates a critical role of JNK in mediating the damage of steatotic hepatocytes. On the other hand, the resistance of KC to damage through activation the survival axis ASK1/p38 MAPK was also detected. 

In the setting of liver transplantation, the treatment with p38 inhibitors decreases reperfusion injury in the liver graft and increases the survival of recipients [39]. However, there are controversies about the role of p38 in liver transplantation [19,39,51,53] and one possible cause of this inconsistency is the difference in the method of analyzing MAPK activity. The study that reported no effect on p38 [19] only examined the activation of MAPKs in liver nuclear extracts, whereas the others examined it in whole cell lysates of liver tissue [39]. Differential activity of subcellular MAPKs has been reported in experimental models of heart I-R [54]. 

In addition to the role of p38 in hepatic I-R injury, it has been demonstrated the involvement of this MAPK in partial hepatectomy under I-R, which is frequently present in clinical practice of liver surgery [45,46]. Under these conditions, p38 was a key mechanism in the benefits of Angiostensin (Ang) II receptor antagonists on liver regeneration in both steatotic and non-steatotic livers. Thus, strategies that increased p38 could improve liver regeneration. In addition, when p38 was inhibited, the benefits of Ang II antagonists on liver regeneration disappeared [45].

In elderly, proinflammatory events occur in the liver, which are responsible for hepatosteatosis development. In an experimental model with ageing mice fed on a high fat diet (HFD) to induce hepatic steatosis, together with tumor necrosis factor alpha (TNF-α) and interleukin (IL)-6, the expression of p-p38 was associated with the proinflammatory response in HFD-fed ageing mice [55]. In elderly, it has been suggested that DIKTNKPVIF bioactive peptide and its parent protein hydrolysate could regulate the inflammation associated with hepatosteatosis development and progression [56]. Such treatment not only enhances lipid metabolism through AMP-activated protein kinase (AMPK) activation but also alleviates hepatic proinflammatory response through attenuating expression of p38 MAPK, TNF-α and IL-6 [56]. All of these observations suggest the potential involvement of MAPKs in elderly animals undergoing hepatic I-R.

**Figure 1:**
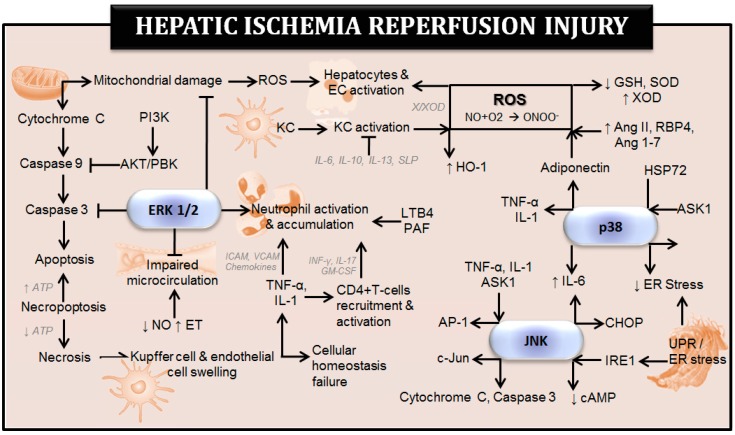
Effect of mitogen-activated protein kinases (MAPKs) on the mechanisms involved in hepatic ischemia-reperfusion injury. Ang: angiotensin; AP-1: activating protein; cAMP: cyclic adenosine monophosphate; Cyt c: cytochrome c; EC: endothelial cell; ERK 1/2: extracellular signal regulated kinases 1/2; ET: endothelin; GM-CSF: granulocyte-macrophage colony-stimulating factor; GSH: glutathione; HO-1: heme oxygenase-1; HSP72: heat shock protein 72; ICAM: intracellular cell adhesion molecule; IL: interleukin; JNK: c-jun N-terminal kinase; KC: kupffer cell; LTB4: leucotriene B4; NO: nitric oxide; ONOO-: peroxynitrite; PAF: platelet activating factor; PBK: protein kinase B; PI3K: phosphoinositide 3-kinase; RBP4: retinol binding protein 4; ROS: reactive oxygen species; SLPI: secretory leukocyte protease inhibitor; SOD: superoxide dismutase; TNF-α: tumor necrosis factor-alpha; VCAM: vascular cell adhesion molecule; X/XOD: xanthine/xanthine oxidase.

Figure 1 shows the effects of MAPKs on some of the mechanisms and cell types involved in hepatic I-R injury. The pathophysiology of hepatic I-R has been previously discussed in different reviews [1,31]. 

#### 2.1.2. Therapeutic Implications of p38 in Hepatic I-R 

The recent research focused on potential regulators of p38 and their therapeutic implications have been shown in steatotic and non-steatotic livers (Table 1 and Table 2, respectively). Inhibitors targeting the p38 pathway have been developed, and preliminary preclinical data suggests that they exhibit anti-inflammatory activity [43,57]. An additional advantage of p38 inhibitors is the possibility that such compounds can be used as an additive to a cold organ preservation solution. A report indicated that addition of a p38 MAPK inhibitor to a cold preservation solution, without administering it to recipients during reperfusion, is sufficient to improve graft viability and the survival rate of recipients [39]. In addition to p38 inhibitors, other drugs that have been used as pharmacological preconditioning are able to prevent p38 effects. This is the case of drugs such as olprinone, all-tras retinoic acid and epidermal growth factor (EGF). All of these reduce p38 activation in livers and the injurious effects of I-R [44,54,57]. Further studies will be required to elucidate whether such pharmacological strategies aimed at regulating p38, could be of clinical interest to protect the liver against I-R injury.

Experimental data indicate that atrial natriuretic peptide (ANP) leads to the activation of p38, which leads to hepatocyte cytoskeletal changes by increasing the hepatocyte, F-actin content in liver [51]. It is known that F-actin is reduced in liver following ischemia, resulting in the loss of cell integrity and cytoplasmic transport in the liver, causing damage to organelles and changes in cell morphology [58]. Beneficial effects of strategies aimed at activating p38 such as Ang II receptor antagonists on hepatic regeneration have been also demonstrated in steatotic and non-steatotic livers in hepatectomy under I-R [45]. Since in clinical situations partial hepatectomy under I-R is usually performed to prevent the blood loss, therapies aimed at trigger p38 activation could be of interest to favor cell growth, particularly in the case of steatotic livers. 

### 2.2. JNK in Hepatic I-R and Their Therapeutic Implications

#### 2.2.1. JNK in Hepatic I-R

JNK is activated in response to stress and inflammatory stimuli [18,37,95]. Numerous studies using experimental models of either warm or cold ischemia have demonstrated an injurious role of JNK activation in I-R injury in steatotic and non-steatotic livers [34,40,47,72,74,75,78,80,95,96,97,98,99,100,101,102,103]. Experimental data indicate that JNK is activated by TNF-α and IL-1, major mediators of hepatic I-R injury. In fact, higher levels of IL-1β were responsible for the exacerbated damage detected in steatotic livers following I-R [104]. Hepatic JNK activation is also associated with inflammation and apoptosis as evidenced by caspase 3 activation and cytochrome c release in liver surgery [96,102]. In our view, the treatment with ROS inhibitors might offer a new way of preventing the deleterious effects of JNK in hepatic I-R. Indeed, JNK activation is induced by mitochondrial ROS generation under I-R conditions [35] (Figure 1). All of these suggest that inhibition of JNK could be beneficial in reducing hepatic I-R injury and in line with this, many studies demonstrated that pharmacological JNK inhibitors attenuated hepatic I-R injury [40,96,102,103]. Only one study in warm ischemia showed the opposite effect since the treatment with a selective inhibitor of JNK increased hepatic injury [96]. Differences in the experimental model of hepatic I-R (warm ischemia with or without hepatectomy) and the dose and administration route of the JNK inhibitor, could explain at least partially, the different data reported in the literature on the role of JNK in hepatic I-R injury in steatotic and non-steatotic livers (Table 3 and Table 4, respectively). Therefore, it is not easy to conclude that JNK inhibition protects the liver against I-R injury [96].

Recently it has been demonstrated that the activation of JNK was associated with impaired liver regeneration in steatotic and non-steatotic livers undergoing partial hepatectomy under I-R [46]. This is opposite to other studies indicating that in regenerating livers following partial hepatectomy without I-R, JNK phosphorylates activating protein 1 (AP-1) at its c-Jun activation domain, which result in transcription of immediate early genes of growth [105]. It should be considered that the role of JNK in hepatic regeneration could be different depending on the surgical conditions evaluated (hepatectomy with or without I-R), as it has been previously demonstrated for other mediators involved in hepatic I-R injury such as Ang II [45,106,107,108]. Considering that in clinical situations, partial hepatectomy under I-R is usually performed to control bleeding during parenchymal dissection, pharmacological strategies aimed at inhibiting JNK might be more appropriated in the clinical surgery of hepatic resections.

#### 2.2.2. Therapeutic Implications of JNK in Hepatic I-R

Preliminary clinical studies indicate the potential benefits of JNK inhibitors in the treatment of fulminant hepatitis and hepatocellular carcinoma in clinical settings [37]. The above discussion on the effect of specific inhibitors of JNK on hepatic I-R injury highlights the possibility and benefit of using JNK inhibitors clinically in the setting of hepatic I-R. Considering the regulation in JNK induced by melatonin and the low potential of harmful side effects of melatonin, this drug is an attractive modality for treating the injurious effects derived from hepatic I-R [34]. Similarly, to melatonin, strategies based on administration of cyclopamine, d-α-tocopherol, ANP or tauroursodeoxycholic acid (TUDCA) could be used as JNK regulators in hepatic I-R injury, but further studies will be required to elucidate whether they are appropriate in clinical practice [38,46,93,99] (Table 3 and Table 4). It should be considered that few studies have been reported on the pharmacological modulation of JNK in steatotic livers. In addition, such studies have been mainly reported on normotermic conditions and without hepatic resection, a surgical condition, commonly used in clinical practice. Furthermore, it should be noted that some of the modulators included in the Table 2, such as NQDI, melatonin, propylene glycol alginate sodium sulfate, remifentanil, beraprost sodium, among others, are not specific regulators of JNK activity. Preclinical studies on pharmacological strategies regulating JNK activity in steatotic liver transplantation will be required if the aims is to extrapolate the preclinical results to clinical practice.

### 2.3. ERK in Hepatic I-R and Their Therapeutic Implications

#### 2.3.1. ERK in Hepatic I-R

ERK1/2, also referred to as p44 and p42 MAP kinases, are ubiquitously expressed kinases and are mainly activated by proliferative stimuli. Hormones and growth factors such as the insulin-like growth factor (IGF), platelet-derived growth factor (PDGF) or EGF are the major regulators of ERK 1/2, but it also may be activated by cytokines, proteins of extracellular matrix such as fibronectin and collagen, glucose and ROS [41,42,110]. Although activation of ERK 1/2 has been mainly associated with cell proliferation and differentiation, there are studies indicating their potential involvement in hepatic I-R injury [7,46,50,80,95,97,98,99,100,111,112,113,114] (Table 5 and Table 6, Figure 1). However, a specific activator or inhibitor of ERK has not been used in any of these studies. Activation of ERK 1/2 has showed to play injurious effects in warm hepatic I-R in both normal and suboptimal livers such as cholestatic livers. These reports indicated that ERK 1/2 was associated with neutrophil infiltration, and both cell death forms, necrosis and apoptosis [7,95,97,98,99]. One study in non-steatotic livers undergoing warm I-R injury have indicated that activation of ERK 1/2 is associated with enhanced protection against hepatic I-R injury, which was related with improved vascular dysfunction and endothelial cell integrity [111]. The design of specific ERK regulators will be required to elucidate whether ERK might be a potential therapeutic target in warm hepatic I-R. Differential effects of ERK 1/2 mentioned under warm hepatic ischemia conditions, have also been reported in the context of liver transplantation. Thus, it seems that the duration of both ischemia and reperfusion affects the cellular response elicited by ERK 1/2 activation in hepatic I-R injury. For instance, ERK 1/2 phosphorylation was associated with protection in liver grafts preserved until 6 hours, whereas it was related with injurious effects in liver grafts preserved for more than 24 hours [50,112,113,114,115]. In line with this, cell signaling, and response induced by ERK 1/2 is dependent on the degree of severity of I-R [14].

#### 2.3.2. Therapeutic Implications of ERK in Hepatic I-R

As previously mentioned in the case of JNK or p38, due to the lack of studies evaluating the effects of a specific activator or inhibitor of ERK 1/2 in hepatic I-R, it is unclear whether ERK 1/2 could be a potential therapeutic target in hepatic I-R injury. However, it has been demonstrated that protection against I-R injury afforded by several pharmacological strategies in steatotic and non-steatotic livers were associated with changes in ERK activation [7,46,50,80,95,97,98,99,100,111,112,113,114].

Cyclopamine, melatonin, sotraustaurin, erythropoietin and IL-1 receptor antagonist are treatments that induce a decrease in the activation of ERK1/2, and a reduction in necrosis and apoptosis, thus protecting against hepatic I-R injury [95,98,99,113,114]. Notoriously, erythropoietin has showed to have the potential to be used as a therapeutic strategy specific for steatotic livers undergoing transplantation, since this drug was used as an additive in preservation solution and this ameliorated cold ischemic injury only in steatotic livers [114]. However, it should be mentioned that neither erythropoietin nor angiotensin HH receptor antagonist, both used in liver transplantation, are specific regulation of ERK activity. Strategies based on the treatment of either, sphinganine-1-phosphate, Ang II receptor antagonists, clotrimazole, TUDCA or cardiotrophin-1 showed that activation of ERK 1/2 is strongly associated with enhanced protection against several forms of injury in hepatic I-R including necrosis and apoptosis [46,50,80,99,112]. Of these treatments, TUDCA could be of interest in clinical situations involving partial hepatectomy under I-R, because activation of ERK 1/2 induced by this drug was not only associated with reduced hepatic damage but also with an improvement in liver regeneration in steatotic and non-steatotic livers [46].

Although all these drugs mentioned above can regulate ERK 1/2 activation in the context of hepatic I-R, they have additional properties and modulate other action mechanisms different to ERK 1/2. Therefore, these experiments do not formally demonstrate that ERK is really an appropriate therapeutic target in hepatic I-R injury. The design of drugs that specifically regulate ERK would be required to elucidate the relevance of ERK as a possible therapeutic target in hepatic I-R injury. Undoubtedly more exhaustive research is needed to translate the usefulness of ERK regulation in hepatic I-R injury from the laboratory to clinical application.

## 3. Role of MAPKs in the Clinical Practice of Hepatic I-R Injury Using Steatotic and Non-Steatotic Livers

There are no investigations about therapeutic strategies (pharmacological or surgical) that modulate MAPK in patients, with the purpose of reducing hepatic I-R injury or the detrimental effects of NAFLD. To the best of our knowledge, there is only one report in the literature in which it was shown that the hypothermic perfusion machine reduced the expression of MAPK (without specifying which) at the beginning of reperfusion, in marginal liver grafts from humans subjected to ex vivo reperfusion. It should be mentioned that the benefits of the hypothermic perfusion machine were not conclusive, and authors of the study recommended continuing to optimize such a preservation method (Table 7).

## 4. Involvement of MAPKs in the Benefits of PC in Hepatic I-R

MAPKs can provide a protective feature through the use of ischemic preconditioning (PC). This surgical strategy is a technique whereby liver is rendered resistant to the damaging effects of I-R by prior exposure to a brief period of vascular occlusion [72]. PC can be either applied directly to the target organ [120] or remotely to a distant vascular bed [121]. The benefits of PC observed in experimental models of hepatic warm and cold ischemia [122,123] prompted human trials of PC. The benefits of PC have been evidenced in patients submitted to partial hepatectomy. Of clinical interest, this surgical strategy protects both steatotic and non-steatotic livers [124]. In our view, PC could resolve, at least partially, the lack of liver grafts available for transplant since it can improve the post-operative outcome of liver grafts from extended criteria donors. However, different results have been reported on the effects of PC in the clinical practice of liver transplantation [125,126]. To explain the higher benefits of ischemic PC in hepatic resections compared with those obtained in a clinical liver transplantation, one has to consider that brain dead is absent in patients undergoing hepatic resections whereas liver grafts are excised from brain dead patients in a clinical liver transplantation. In this sense, it should be noted that in an experimental liver transplantation from cadaveric donors, brain death abrogates the benefits of ischemic PC in steatotic and non-steatotic liver transplantation [127,128]. Indeed, in the setting of liver transplantation, the inflammatory response induced by brain dead, present in the liver before the induction of ischemic PC, would interact with various mechanistic aspects of PC and block the eventual ischemic PC response. Thus, in the setting of clinical transplantation, the combination of PC with pharmacological treatments such as acetylcholine (ACH), which reduced the deleterious effect of brain dead before PC application, would be required. We have demonstrated that the protection conferred by the combination of the PC and ACH treatment was stronger than those obtained by these strategies separately. Interestingly, since the ischemic period and pathophysiology is similar in partial hepatectomy and living donor liver transplantation, PC could reduce damage and improve liver regeneration failure, a relevant risk factor in living donor liver transplantation [129]. In fact, Barrier et al. has reported the benefits of PC in the clinical practice of living related liver transplantation [130].

**Figure 2:**
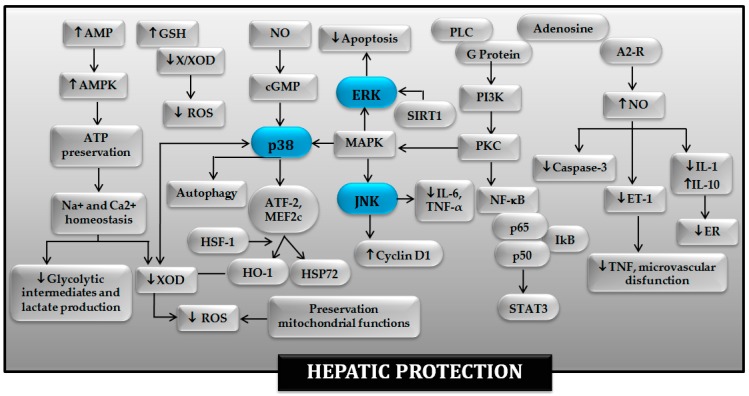
Involvement of MAPKs in the protective mechanisms of ischemic preconditioning in the hepatic ischemia-reperfusion injury. A2-R: adenosine 2 receptor; AMP: adenosine monophosphate; AMPK: AMP-activated protein kinase; ATF-2: Activating transcription factor-2; ATP: adenosin triphosphate; cGMP: Guanosine 3′,5′-cyclic monophosphate; ER: Endoplasmic reticulum; ERK: Extracellular signal-regulated protein kinases; ET-1: endothelin-1; GSH: Glutathione; HO-1: heme oxygenase-1; HSF-1: Heat shock transcription factor-1; HSP72: heat-shock protein 72; IL: interleukin; JNK: jun N-terminal kinase; MAPK: mitogen-activated protein kinase; MEF2c: myocyte enhancer factor-2; NF-κB: factor nuclear factor-kappa B; NO: Nitric oxide; PI3K: Phosphatidylinositol 3-kinase; PKC: protein kinase C; PLC: phospholipase C; ROS: Reactive oxygen species; SIRT1: sirtuin-1; STAT3: signal transducer and activator of transcription-3; TNF: tumor necrosis factor; X/XOD: xanthine/xanthine oxidase.

The complex interaction between the different signaling pathways induced by PC, namely protein kinase C (PKC), MAPK and p38, has been previously discussed in excellent manuscripts [131,132,133,134] and summarized in Figure 2. In non-steatotic liver, it is possible that NF-κB and p38 MAPK-regulated transcription factors like ATF-2 and myocyte enhancer factor-2 (MEF2C) might be responsible for inducing the expression of protective genes (Figure 2) [40]. The activation of p38 and JNK-1 induced by PC is associated with increased cyclin D1 expression and entry into the cell cycle [74]. Induction of cyclin D1 is one of the earliest and most pivotal steps in the pathway of resting cells to enter the cell cycle. In addition to this, activation of p38 has been considered to be a crucial mechanism of hepatoprotection in the context of different pharmacological strategies aimed at mimicking PC effects, including agonists of the adenosine A2 receptor, carbon monoxide (CO), nitric oxide (NO) and ANP [135]. In addition, autophagic flux is enhanced by liver PC, since endothelial nitric oxide synthase (eNOS)-derived NO activates autophagy via phosphorylation of p38 MAPK [71]. The induction of PC prior to warm I-R is able to activate heme oxygenase-1 (HO-1), an enzyme with antioxidant properties, which is constitutively expressed in hepatocytes, endothelial cells and stellate cells [136]. HO-1-dependent signaling has been implicated in remote PC, where regulation of p38 MAPK and ERK 1/2 leads to the alleviation of the hepatic IR injury [137]. It has been also suggested that activation of the ERK 1/2 pathway plays a vital role in the hepatocyte protection induced by PC [138]. On the contrary, another report indicates that intracellular signals involved in ATP-dependent preconditioning increases hepatocyte resistance to hypoxia by down-modulating ERK1/2-mediated signals [135]. Further investigation will be required to clarify the role of ERK 1/2 in the benefits of PC on hepatic I-R. Regarding the molecular basis of PC in liver hepatectomy, its beneficial effects have been shown to be linked to better ATP recovery, nitric oxide production, antioxidant activities and endoplasmic reticulum adaptation. All of this limited mitochondrial damage and apoptosis. In addition the ERK1/2 and p38 MAPK activation induced by PC favors liver regeneration [88]. Furthermore, PC can initiate hepatocyte proliferation action in partial hepatectomy under vascular occlusion by a signaling mechanism involving p-JNK and TNF-α/IL-6 signal pathway [139].

There are very few studies addressing the molecular basis of PC in steatotic livers. In contrast with non-steatotic livers, PC reduces MAPK activation (JNK and p38), and this is associated with protection against hepatic I-R injury in the presence of steatosis [40,48]. The involvement of sirtuin-1 (SIRT1) induction in the benefits of PC on normothermic hepatic conditions has been reported [140]. Thus, SIRT1 inhibition decreased the expression of p-ERK and augmented p-p38 protein levels [140]. ERK activation during PC protects against I-R injury in steatotic livers, by inhibiting apoptosis [141], whereas treatment with a p38 activator abolished the benefits of PC on hepatic damage [40]. Nonetheless, further investigation will be required to address the involvement of MAPKs in the protective effects of PC in steatotic livers undergoing liver transplantation.

## 5. Conclusions and Perspectives

MAPKs have a dual role; they are involved in the benefits of strategies such as ANP or PC on hepatic I-R injury, yet they also act as a catalyst for liver damage. One possible reason for this could be that specific MAPK isoforms might play different roles. This point needs to be addressed under hepatic I-R conditions if our aims are the regulation of MAPK activity to prevent hepatic I-R injury. For instance, the activation of p38α isoform promotes cell death in the heart whereas p38β has anti-apoptotic effects. This should be also the case for JNK isoenzymes.

The response of steatotic and non-steatotic livers to MAPK stimulation might differ and involve different signal transduction pathways that are at present marginally understood. This could entail that some strategies based on MAPK modulation could be specific for each type of the liver. For this reason, further research is required to clarify the differences about the role of MAPK in hepatic I-R injury between steatotic and non steatotic livers, in order to find the most appropriate MAPK modulator to reduce I-R injury in each type of liver.

Further research is required to select drugs that regulate MAPK with minimal side effects before such approaches can be translated into treatments for human disease. To avoid potential side effects of MAPK pharmacological modulators, strategies such as the induction of PC that regulate MAPK, alone or combined with pharmacological treatments, could be of clinical interest in human liver resections and liver transplantation in both steatotic and non-steatotic livers. PC is easy to apply, inexpensive and does not require the use of drugs with potential side effects. One disadvantage of PC is that it requires a period of pre-ischemic manipulation for organ protection.

Whether the mentioned MAPK regulators discussed herein can be translated into clinical practice remains unknown, but further researches are required to optimize their use (e.g., dose, pharmacokinetics, etc.). Such approaches based in regulating MAPKs have the potential to improve the post-operative outcomes in hepatic resections and to increase the number of organs suitable for transplantation, since they may improve the outcomes of marginal grafts that would not otherwise have been transplanted, opening up new possibilities for steatotic liver transplants.

## Figures and Tables

**Table 1:** Effect of strategies that regulate p38 in experimental models of hepatic ischemia- reperfusion using steatotic livers.

Experimental Model		Changes in p38 Induced by I-R	Treatment with p38 Modulators	Dose and Administration	Effect of p38 Modulators
Species	Surgical Procedure				
Mice	Partial Warm I-R I: 45 min-R: 120 min [59]	↑ p38	NQDI-1	0.25 mg/kg, i.p., twice a week before surgery	↓ p38 phosphorylation, damage
Rats	Partial Warm I-R I: 60 min-R: 30 min [40]	↑ p38	SB203580	1 mg/kg, i.p., 24 h before I-R	↓ p38 phosphorylation, damage ↑ HSP72
	Partial Warm I-R I: 60 min-R: 30 min [40]	↑ p38	Anisomycin PC	0.1 mg/kg, i.p., 24 h before PC	↑ p38 phosphorylation, damage
Mice	Total Warm I-R I: 15 min-R: 3 h [52]	↑ p38	Astaxanthin	25 mg/kg, oral, 48 h, 24 h and 40 min before I-R	↓ p38 phosphorylation, damage apoptosis
Rats	I-R with Partial (70%) Hepatectomy I: 60 min-R: 24 h [44]	↑ p38	Anisomycin	0.1 mg/kg, na, 24 h before surgery	No activity changes in p38
	I-R with Partial (70%) Hepatectomy I: 60 min-R: 24 h [44]	↑ p38	SB203580	1 mg/kg, na, 24 h before surgery	↓ p38 phosphorylation, damage
	I-R with Partial (70%) Hepatectomy I: 60 min-R: 24 h [44]	↑ p38	EGF	100 µg/kg, na, before surgery every 8 h for 3 doses	↓ p38 phosphorylation
	I-R with Partial (70%) Hepatectomy I: 60 min-R: 24 h [45]	↑ p38	Losartan PD123319	Losartan: 5 mg/kg, oral, 24 and 1.5 h before surgery PD123319: 30 mg/kg, i.v., 5 min before surgery	↓ Damage ↑ p38 phosphorylation, regeneration
	I-R with Partial (70%) Hepatectomy I: 60 min-R: 24 h [46]	↑ p38	TUDCA	50 mg/kg, na, before surgery	↓ mitochondrial damage, ER stress ↑ p38 phosphorylation, regeneration

Specific p38 Inhibitor: SB203580, PD123319. Non-selective p38 Modulators: Anisomycin; Astaxanthin; EGF; Losartan; NDQI-1: NQDI-1–2,7-Dihydro-2,7-dioxo-3H-naphth-o[1,2,3dequin-oline-1-carboxylic acid ethyl ester]; PC; TUDCA.

**Table 2:** Effect of strategies that regulate p38 in experimental models of hepatic ischemia- reperfusion using non-steatotic livers.

Experimental Model		Changes in p38 Induced by I-R	Treatment with p38 Modulators	Dose and Administration	Effect of p38 Modulators
Species	Surgical Procedure				
Mice	Partial Warm I-R I: 45 min-R: 2, 8, 24 h [60]	↑ p38	Beraprost sodium	50, 100 μg/kg/d, oral, 7 days before I-R	↓ p38 phosphorylation, damage, apoptosis, autophagy
	Partial Warm I-R I: 45 min-R: 24 h [61]	↑ p38	Propylene glycol alginate sodium sulfate	25, 50 mg/kg, i.p., 1 h before surgery	↓ p38 phosphorylation, damage, apoptosis, autophagy, inflammation
	Partial Warm I-R I: 60 min-R: 1, 3, 6, 12, 24 h [62]	↑ p38	5Z-7-oxozeaenol	5 mg/kg, i.p., 30 min before I-R	↓ p38 phosphorylation, NF-κB, damage, inflammation
	Partial Warm I-R I: 60 min-R: 1, 3, 6, 12 h [63]	↑ p38	Nilotinib	30 mg/kg, oral, 12 and 2 h before I-R	↓ p38 phosphorylation, damage, inflammatory cytokines
	Partial Warm I-R I: 60 min-R: 1, 6, 24 h [64]	↑ p38	5Z-7-oxozeaenol	Adenovirus vectors, i.v., 4 weeks before I-R	↓ p38 phosphorylation, damage, inflammation
	Partial Warm I-R I: 60 min-R: 1, 4, 24 h [65]	↑ p38	Methyl-beta-cyclodextrin	10, 25, 50 mg/kg, i.p., 48 and 24 h before I-R	↑ p38 phosphorylation, damage, apoptosis
	Partial Warm I-R I: 60 min-R: 2, 4, 8, 12, 24 h [66]	↑ p38	SIRT6 ^-/-^ KO	na	↓ p38 phosphorylation, damage, NF-κB
	Partial Warm I-R I: 60 min-R: 2, 4, 24 h [67]	↑ p38	Astaxanthin	30, 60 mg/kg, oral, 14 consecutive days before I-R	↓ p38 phosphorylation, damage, apoptosis, ROS, autophagy
	Partial Warm I-R I: 60 min-R: 3, 6, 12, 24 h [68]	↑ p38	RC-3095	3 mg/kg, i.p., at the time of reperfusion	↓ p38 phosphorylation, damage, inflammation
	Partial Warm I-R I: 60 min-R: 6 h [69]	↑ p38	Schisantherin A	200 mg/kg/d, oral, 5 days	↓ p38 phosphorylation, damage
	Partial Warm I-R I: 60 min-R: 6 h [70]	↑ p38	PC	10 min ischemia, 10 min reperfusion	↓ p38 phosphorylation, damage
	Partial Warm I-R I: 60 min-R: 6, 18 h [71]	↑ p38	MK2 ^-/-^ KO	na	↓ p38 phosphorylation, damage, ROS production
	Partial Warm I-R I: 90 min-R: 90 min [72]	No activity changes in p38	PC	10 min ischemia, 10 min reperfusion	↓ Damage, NF-κB ↑ p38 phosphorylation
	Partial Warm I-R I: 90 min-R: 1, 4, 8 h [73]	↑ p38	IL-33 recombinant	5, 10 µg/mice, i.p., 16 and 1 h before I-R	↓ Damage ↑ p38 phosphorylation, NF-κB, cyclin D1, Bcl-2
	Partial Warm I-R I: 90 min-R: 2, 24, 44 h [74]	↑ p38	TNF-α + PC (I-R: 10 min)	1, 5 µg/kg, na, 30 min before I-R	↓ Damage ↑ p38 phosphorylation, NF-κB, STAT3, cyclin D1, cdk4
	Partial Warm I-R I: 90 min-R: 2, 24, 44 h [75]	↑ p38	TNF ^-/-^ KO	na	↓ Damage ↑ p38 phosphorylation
Rats	Partial Warm I-R I: 30 min-R: 3 h [76]	↑ p38	Melatonin	10 mg/kg, i.v., 15 min before I & 10 min before R	↓ p38 phosphorylation, damage
	Partial Warm I-R I: 30 min-R: 72 h [77]	↑ p38	Rebamipide	60, 100 mg/kg, i.p., after reperfusion and for 2 days	↓ p38 phosphorylation, damage, HMGB1, caspase-3 ↑ ATP
	Partial Warm I-R I: 60 min-R: 30 min [40]	↑ p38	SB203580	1 mg/kg, i.p., 24 h before I-R	↓ p38 phosphorylation, damage ↑ HSP72
	Partial Warm I-R I: 60 min-R: 30 min [40]	↑ p38	Anisomycin PC	0.1 mg/kg, i.p., 24 h before PC	↑ p38 phosphorylation, damage
	Partial Warm I-R I: 60 min-R: 3 h [78]	↑ p38	Olprinone	20 µg/kg, i.v., 5 min before reperfusion	↓ p38 phosphorylation, damage, NF-κB, IL-6, TNFα, ICAM-1 ↑ cAMP
	Partial Warm I-R I: 60 min-R: 4 h [79]	↑ p38	Cryptotan- shinone	50 mg/kg/d, i.p., 7 days before I-R	↓ p38 phosphorylation, damage, apoptosis
	Partial Warm I-R I: 60 min-R: 6 h [80]	↑ p38	Clotrimazole	50 mg/kg, i.p., 3 days before I-R	No activity changes in p38 ↓ Damage, apoptosis
	Partial Warm I-R I: 60 min-R: 24 h [81]	↑ p38	All-*tras* retinoic acid	15 mg/kg/d, i.p., 14 days before I-R	↓ p38 phosphorylation, damage
	Partial Warm I-R I: 60 min-R: 120 min [82]	↑ p38	Anisomycin	0.5 µg/ml, i.v., 60-70 min in 100 total min of I-R	↑ p38 phosphorylation, damage, GSH/GSSG redox system
	Partial Warm I-R I: 60 min-R: 5 days [83]	No activity changes in p38	PC	10 min ischemia, 15 min reperfusion	↑ p38 phosphorylation
	Partial Warm I-R I: 60 min-R: 7 days [84]	No activity changes in p38	Acanthopanax divaricatus vat. albeofructus	600 mg/kg, oral, 2 weeks before I-R	↓ Damage ↑ p38 phosphorylation, IL-10
	Partial Warm I-R I: 90 min-R: 6, 11, 24, 60 h [85]	↑ p38	HDACi valproic & suberoylanilide hydroxamic acids	Valproic acid: 300 mg/kg, i.p., 24, 12 and 0 h before I-R, Suberoylanilide: 60 mg/kg, i.p., 24 and 0 h before I-R	↓ p38 phosphorylation ↑ Damage
	Partial Warm I-R I: 120 min-R: 1, 3, 6 h [47]	↑ p38	Z-Asp-cmk	50 mg/ml, i.v., before I-R	↓ Damage, apoptosis ↑ p38 phosphorylation
Mice	Total Warm I-R I: 45 min-R: 2, 8, 24 h [86]	↑ p38	Salidroside	20 mg/kg, i.p., 2 h before I-R	↓ p38 phosphorylation, damage, apoptosis, autophagy
Rats	Total Warm I-R I: 30 min-R: 30, 60, 90, 120 min [33]	↑ p38	FR167653	0.1 mg/kg/h, i.v., 30 minutes before I to 2 h after R	↓ p38 phosphorylation, damage, IL-1β, TNF-α
Pig	Total Warm I-R I: 120 min-R: 2 h [58]	↑ p38	ANP	0.1 μg/kg/min, i.v., 30 min before I to the end of the R	↓ Damage, TNF-α, NF-κB ↑ p38 phosphorylation
Rats	I-R with Partial (70%) Hepatectomy I: 15 min-R: 1 h [87]	↑ p38	Inchinkoto	1, 2 g/kg, na, 3 days before I-R	↓ p38 phosphorylation, damage ↑ Survival
	I-R with Partial (70%) Hepatectomy I: 30 min-R: 3, 9, 18, 24, 30, 48 h [88]	No activity changes in p38	PC	10 min ischemia, 5 min reperfusion	↓ Damage, apoptosis ↑ p38 phosphorylation, mitochondrial integrity
	I-R with Partial (70%) Hepatectomy I: 60 min-R: 24 h [44]	↑ p38	Anisomycin	0.1 mg/kg, na, 24 h before I-R	No activity changes in p38
	I-R with Partial (70%) Hepatectomy I: 60 min-R: 24 h [44]	↑ p38	SB203580	1 mg/kg, na, 24 h before I-R	↓ p38 phosphorylation, damage
	I-R with Partial (70%) Hepatectomy I: 60 min-R: 24 h [44]	↑ p38	EGF	100 µg/kg, na, before surgery every 8 h for 3 doses	↓ p38 phosphorylation, damage
	I-R with Partial (70%) Hepatectomy I: 60 min-R: 24 h [45]	↑ p38	Losartan PD123319	Losartan: 5 mg/kg, oral, 24 and 1.5 h before surgery. PD123319: 30 mg/kg, i.v., 5 min before surgery	↓ Damage ↑ p38 phosphorylation, regeneration
	I-R with Partial (70%) Hepatectomy I: 60 min-R: 24 h [46]	↑ p38	TUDCA	100 mg/kg, na, before surgery	↓ Mitochondrial damage, ER stress ↑ p38 phosphorylation, regeneration
Rats	Liver transplantation Cold I: 30 min-R: 3, 6, 24 h [89]	↑ p38	5′-Methylthio- adenosine	96 μmol/kg, i.p., 30 min before harvest	↓ p38 phosphorylation, damage, NF-κB, inflammation
	Liver transplantation Cold I: 1 h-R: 24 h [90]	↑ p38	Losartan	5 mg/kg, oral, 24 and 1 h before surgery	↓ p38 phosphorylation, damage ↑ SIRT1
	Liver transplantation Cold I: 18 h-R: 1, 3, 6, 24, 48 h [91]	↑ p38	Carbon monoxide	100 ppm in recipient, 1 h before surgery and 1, 3, and 6 h after surgery	No activity changes in p38 ↓ Damage
	Liver transplantation Cold I: 24 h-R: 2 h [51]	↑ p38	ANP	200 nmol/L, na, 10 min after perfusion	↓ Damage, alterations of the hepatocyte cytoskeleton ↑ p38 phosphorylation
	Liver transplantation Cold I: 24 h-R: 6, 24 h [92]	↑ p38	CS-1 peptide	500 µg/rat, i.v, before cold storage and reperfusion. 1 mg/rat, i.p., 1 h after transplant in recipients	↓ p38 phosphorylation, damage, TNF-α, INF-γ, iNOS, leukocyte recruitment ↑ Survival
	Liver transplantation Cold I: 30 h-R: 6, 24 h [39]	↑ p38	FR167653 as additive	100 ml/kg, in perfused, before removal the liver	↓ p38 phosphorylation, damage ↑ Survival
Rats	Isolated Liver Perfused Cold I: 24 h-R: 45 min [51]	↑ p38	SB203580 + ANP	SB203580: 2 µmol/L, na, after perfusion. ANP: 200 nmol/L, na, 10 min after perfusion.	↓ p38 phosphorylation, Hsp27 ↑ Damage
	Isolated Liver Perfused Cold I: 24 h-R: 2 h [93]	↓ p38	ANP	200 nM added to the perfusate for 20 min	↓ Damage ↑ p38 activity
	Isolated Liver Perfused Cold I: 24 h-R: 2 h [94]	↑ p38	SB203580	25 mg/kg, oral, 1 h before harvest and ex vivo perfusion with 20 µmol/L plus CO	↓ Damage, apoptosis

Specific p38 Inhibitor: SB203580, FR167653, PD123319. Non-selective p38 Modulators: 5′-Methylthioadenosine; 5Z-7-oxozeaenol: TAK1 inhibitor; Acanthopanax divaricatus vat. Albeofructus; All-tras Retinoic Acid; Anisomycin; ANP; Astaxanthin; Beraprost sodium; Carbon monoxide; Clotrimazole; Cryptotanshinone; CS-1; EGF; HDACi valproic acid; IL-33 recombinant; Inchinkoto; Losartan; Melatonin; Methyl-beta-cyclodextrin: Caveolae disruptor; MK2; Nilotinib; Olprinone; preconditioning (PC); Propylene glycol alginate sodium sulfate; RC-3095: selective gastrin-releasing peptide receptor (GRPR) antagonist; Rebamipide; Salidroside; Schisantherin A; SIRT6; Suberoylanilide hydroxamic acid; TNF-α; TUDCA, Z-Asp-cmk: Z-Asp-2,6-dichorobenzoyloxymethylketone.

**Table 3:** Effect of strategies that regulate JNK in experimental models of hepatic ischemia- reperfusion using steatotic livers.

Experimental Model		Changes in JNK Induced by I-R	Treatment with JNK Modulators	Dose and Administration	Effect of JNK Modulators
Species	Surgical Procedure				
Mice	Partial Warm I-R I: 45 min-R: 120 min [59]	↑ JNK	NQDI-1	0.25 mg/kg, i.p., twice a week before surgery	↓ JNK phosphorylation, damage
Rats	Partial Warm I-R I: 60 min-R: 30 min [40]	↑ JNK	SP600125	6 mg/kg, s.c., 1 h before I-R	↓ JNK phosphorylation, damage
Mice	Total Warm I-R I: 15 min-R: 3 h [52]	↑ JNK	Astaxanthin	25 mg/kg, oral, 48, 24 h and 40 min before I-R	↓ JNK phosphorylation, damage, apoptosis
Rats	I-R with Partial (70%) Hepatectomy I: 60 min-R: 24 h [46]	↑ JNK	TUDCA	100 mg/kg, na, before surgery	↓ JNK phosphorylation, damage, caspase 12

Specific JNK Inhibitor: SP600125. Non-selective JNK Modulators: Astaxanthin; NDQI-1, NQDI-1–2,7-Dihydro-2,7- dioxo- 3H-naphth-o[1,2,3dequin-oline-1-carboxylic acid ethyl ester]; TUDCA.

**Table 4:** Effect of strategies that regulate JNK in experimental models of hepatic ischemia- reperfusion using non-steatotic livers.

Experimental Model		Changes in JNK Induced by I-R	Treatment with JNK Modulators	Dose and Administration	Effect of JNK MODULATORS
Species	Surgical Procedure				
**Mice**	Partial Warm I-R I: 45 min-R: 2 h [109]	↑ JNK	Remifentanil	30 μg/kg, i.p., 10 min before I-R	↓ JNK phosphorylation, damage
	Partial Warm I-R I: 45 min-R: 2, 8, 24 h [60]	↑ JNK	Beraprost sodium	50, 100 μg/kg/d, oral, 7 days before I-R	↓ JNK phosphorylation, damage, apoptosis, autophagy
	Partial Warm I-R I: 45 min-R: 24 h [61]	↑ JNK	Propylene glycol alginate sodium sulfate	25, 50 mg/kg, i.p., 1 h before surgery	↓ JNK phosphorylation, damage, apoptosis, autophagy, inflammation
	Partial Warm I-R I: 60 min-R: 15 min, 1 h [96]	↑ JNK	SP600125	10 mg/kg, i.p., at the onset of ischemia and 10 mg/kg, s.c., after reperfusion	↓ JNK phosphorylation ↑ Damage, leukocyte infiltration
	Partial Warm I-R I: 60 min-R: 1, 3, 6, 12, 24 h [62]	↑ JNK	5Z-7-oxozeaenol	5 mg/kg, i.p., 30 min before I-R	↓ JNK phosphorylation, damage, inflammation, NF-κB
	Partial Warm I-R I: 60 min-R: 1, 6, 24 h [64]	↑ JNK	5Z-7-oxozeaenol	Adenovirus vectors, i.v., 4 weeks before I-R	↓ JNK phosphorylation, damage, inflammation
	Partial Warm I-R I: 60 min-R: 1, 4, 24 h [65]	↑ JNK	Methyl-beta-cyclodextrin	10, 25, 50 mg/kg, i.p., 48 and 24 h before I-R	↑ JNK phosphorylation, damage, apoptosis
	Partial Warm I-R I: 60 min-R: 2, 4, 8, 12, 24 h [66]	↑ JNK	SIRT6 ^-/-^ KO	na	↓ JNK phosphorylation, damage, NF-κB
	Partial Warm I-R I: 60 min-R: 2, 4, 24 h [67]	↑ JNK	Astaxanthin	30, 60 mg/kg, oral, 14 consecutive days before I-R	↓ JNK phosphorylation, damage, apoptosis, ROS, autophagy
	Partial Warm I-R I: 60 min-R: 6 h [69]	↑ JNK	Schisantherin A	200 mg/kg/d, oral, 5 days	↓ JNK phosphorylation, damage
	Partial Warm I-R I: 60 min-R: 6 h [70]	↑ JNK	PC	10 min ischemia, 10 min reperfusion	No activity changes in JNK ↓ Damage
	Partial Warm I-R I: 90 min-R: 1, 2, 4, 6 h [97]	↑ JNK	CXCL10 ^-/-^ KO	na	No activity changes in JNK ↓ Damage, neutrophil infiltration
	Partial Warm I-R I: 90 min-R: 90 min [72]	No activity changes in JNK	PC	10 min ischemia, 10 min reperfusion	↓ Damage, NF-κB ↑ JNK phosphorylation
	Partial Warm I-R I: 90 min-R: 2, 4 h [108]	↑ JNK	SB203580	shRNA plasmids: 10 μg in 2 ml, i.v., 72 h before I-R	↓ JNK phosphorylation, damage
	Partial Warm I-R I: 90 min-R: 2, 24, 44 h [74]	↑ JNK	TNF-α + PC (I-R: 10 min)	1, 5 µg/kg, na, 30 min before I-R	↓ Damage ↑ JNK phosphorylation
	Partial Warm I-R I: 90 min-R: 2, 24, 44 h [75]	↑ JNK	TNF ^-/-^ KO	na	↓ JNK phosphorylation, damage
**Rats**	Partial Warm I-R I: 30 min-R: 3 h [76]	↑ JNK	Melatonin	10 mg/kg, i.v., 15 min before I & 10 min before R	↓ JNK phosphorylation, damage
	Partial Warm I-R I: 60 min-R: 30 min [95]	↑ JNK	IL-1 RA	100–400 µg/100 g, i.p., 30 min before I and immediately after R	↓ JNK phosphorylation, damage, IL-6, TNF-α
	Partial Warm I-R I: 60 min-R: 30 min [40]	↑ JNK	SP600125	6 mg/kg, s.c., 1 h before I-R	↓ JNK phosphorylation, damage
	Partial Warm I-R I: 60 min-R: 1, 5 h [98]	↑ JNK	Melatonin	10 mg/kg, i.v., 15 min before I & 10 min before R	↓ JNK phosphorylation, damage, inflammatory signaling
	Partial Warm I-R I: 60 min-R: 3 h [78]	↑ JNK	Olprinone	20 µg/kg, i.v., 5 min before reperfusion	↓ JNK phosphorylation, damage, IL-6, TNF-α, NF-κB, ICAM-1 ↑ cAMP
	Partial Warm I-R I: 60 min-R: 4 h [79]	↑ JNK	Cryptotan- shinone	50 mg/kg/d, i.p., 7 days before I-R	↓ JNK phosphorylation, damage, apoptosis
	Partial Warm I-R I: 60 min-R: 6 h [80]	↑ JNK	Clotrimazole	50 mg/kg, i.p., 3 days before I-R	↓ JNK phosphorylation, damage, apoptosis
	Partial Warm I-R I: 60 min-R: 7 days [84]	↑ JNK	Acanthopanax divaricatus vat. albeofructus	600 mg/kg, oral, 2 weeks before I-R	↓ JNK phosphorylation, damage ↑ IL-10
	Partial Warm I-R I: 90 min-R: 1 h [99]	↑ JNK	Cyclopamine	10 mg/kg, i.p., 3 days and 1 h before surgery	↓ JNK phosphorylation, damage, inflammation
	Partial Warm I-R I: 90 min-R: 6, 11, 24, 60 h [85]	↑ JNK	HDACi valproic & suberoylanilide hydroxamic acids	Valproic acid: 300 mg/kg, i.p., 24, 12 and 0 h before I-R, Suberoylanilide: 60 mg/kg, i.p., 24 and 0 h before I-R	↑ JNK phosphorylation, damage
	Partial Warm I-R I: 120 min-R: 1, 3, 6 h [47]	↑ JNK	Z-Asp-cmk	50 mg/ml, i.v., before I-R	↓ Damage, apoptosis ↑ JNK phosphorylation
**Mice**	Total Warm I-R I: 45 min-R: 2, 8, 24 h [86]	↑ JNK	Salidroside	20 mg/kg, i.p., 2 h before I-R	↓ JNK phosphorylation, damage, apoptosis, autophagy
	Total Warm I-R I: 10, 15 min-R: 10, 20, 30 min [38]	↑ JNK	d-α-tocopherol	40 mg/kg, i.p., 2 weeks before surgery	↓ JNK phosphorylation, damage, apoptosis
**Mice**	I-R with Partial (30%) Hepatectomy I: 75 min-R: 1 h [100]	↑ JNK	RAGE ^-/-^ KO	na	↓ JNK phosphorylation, damage, neutrophil infiltration, TNF-α
	I-R with Partial (70%) Hepatectomy I: 45 min-R: 1, 6, 24 h [101]	↑ JNK	17β-Estradiol	1 µg in 100 ml, s.c., 24 h before I-R	↓ JNK phosphorylation ↑ Regeneration, survival
**Rats**	I-R with Partial (30%) Hepatectomy I: 60 min-R: 60 min [103]	↑ JNK	CC0223105 CC0209766 CC-401	3-20 mg/kg, i.v., 15 min before ischemia	↓ JNK phosphorylation, damage, cytochrome c release, apoptosis, ROS ↑ Survival
	I-R with Partial (68%) Hepatectomy I: 30 min-R: 3, 8, 48 h [34]	↑ JNK	Melatonin	50 mg/kg, oral, 2 h before ischemia	↓ JNK phosphorylation, damage, iNOS, leukocyte infiltration ↑ Survival
	I-R with Partial (70%) Hepatectomy I: 30 min-R: 3, 9, 18, 21, 24, 30, 48 h [88]	No activity changes in JNK	PC (I-R: 10-5 min)	10 min ischemia, 5 min reperfusion	↓ JNK phosphorylation, damage, apoptosis ↑ Mitochondrial integrity
	I-R with Partial (70%) Hepatectomy I: 60 min-R: 24 h [46]	↑ JNK	TUDCA	100 mg/kg, na, before surgery	↓ JNK phosphorylation, damage, caspase 12
**Rats**	Liver transplantation Cold I: 18 h-R: 1, 3, 6, 24, 48 h [91]	↑ JNK	Carbon monoxide	100 ppm in recipient, 1 h before surgery and 1, 3, and 6 h after surgery	No activity changes in JNK ↓ Damage
	Liver transplantation Cold I: 30 h-R: 6, 24 h [39]	↑ JNK	FR167653 as additive	100 ml/kg, in perfused, before removal the liver	No activity changes in JNK ↓ Damage ↑ Survival
	Liver transplantation Cold I: 44 h-R: 15 min, 1, 8 h [102]	↑ JNK	CC-401	10 mg/kg, i.v., 10 min before removal the liver and 20 µM in the solution for 44 h	↓ JNK phosphorylation, damage, TNF-α, caspase 3, apoptosis
**Rats**	Isolated Liver Perfused I: 24 h-R: 2 h [93]	↑ JNK	ANP	200 nM added to the perfusate for 20 min	No activity changes in JNK ↓ Damage

Specific JNK Inhibitor: CC0223105, CC0209766, CC-401, SB203580, SP600125. Non-selective JNK Modulators: 17β-estradiol; 5Z-7-oxozeaenol: TAK1 inhibitor; Acanthopanax divaricatus vat. Albeofructus; ANP; Astaxanthin; Beraprost sodium; Carbon monoxide; Clotrimazole; Cryptotanshinone; CXCL10; Cyclopamine; d-α-tocopherol; FR167653; HDACi valproic acid; IL-1 RA receptor antagonist; Melatonin; Methyl-beta-cyclodextrin; Olprinone; PC; Propylene glycol alginate sodium sulfate; RAGE; Remifentanil; Salidroside; Schisantherin A; SIRT6; Suberoylanilide hydroxamic acid; TNF-α; TUDCA; Z-Asp-cmk: Z-Asp-2,6-dichorobenzoyloxymethylketone.

**Table 5:** Effect of strategies that regulate ERK in experimental models of hepatic ischemia- reperfusion using steatotic livers.

Experimental Model		Changes in ERK Induced by I-R	Treatment with ERK Modulators	Dose and Administration	Effect of ERK Modulators
Species	Surgical Procedure				
Mice	Total Warm I-R I: 15 min-R: 3 h [52]	↓ ERK	Astaxanthin	25 mg/kg, oral, 48, 24 h and 40 min before I-R	↓ ERK phosphorylation, damage, apoptosis
Rats	I-R with Partial (70%) Hepatectomy I: 60 min-R: 24 h [46]	↑ ERK	TUDCA	50 mg/kg, na, before surgery	↓ Damage, GSK3β activity ↑ ERK phosphorylation
Rats	Liver transplantation Cold I: 4 h-R: 1, 3, 7 days [115]	↑ ERK	CS-1 peptide	500 µg/rat, i.v., before and during surgery and 1 mg/rat/day, i.v., 3 days in recipients	↓ ERK phosphorylation, damage, IL-2, IFN-γ, T-cell
	Liver transplantation Cold I: 6 h-R: 4 h [50]	No activity changes in ERK	Angiotensin II receptor antagonists	AT1R: 5 mg/kg, oral, 24 and 1.5 h before surgery AT2R: 30 mg/kg, i.v., 5 min before surgery	↓ Damage ↑ ERK phosphorylation, survival
Mice	Isolated liver perfused Cold I: 24 h-R: 2 h [114]	↓ ERK	Erythropoietin as additive	10 IU/mL in solution	↓ ERK phosphorylation, damage

Non-Selective ERK Modulators: Angiotensin II receptor antagonists; Astaxanthin; CS-1 peptide; Erythropoietin; TUDCA.

**Table 6:** Effect of strategies that regulate ERK in experimental models of hepatic ischemia-reperfusion using non-steatotic livers.

Experimental Model		Changes in ERK Induced by I-R	Treatment with ERK Modulators	Dose and Administration	Effect of ERK Modulators
Species	Surgical Procedure				
Mice	Partial Warm I-R I: 30 min-R: 4 h [7]	↑ ERK	Cyclopamine	10 mg/kg/day, na, 14 days before I-R	↓ ERK phosphorylation, damage, Akt, apoptosis
	Partial Warm I-R I: 45 min-R: 2 h [109]	↑ ERK	Remifentanil	30 μg/kg, i.p., 10 min before I-R	↓ ERK phosphorylation, damage
	Partial Warm I-R I: 45 min-R: 24 h [61]	↑ ERK	Propylene glycol alginate sodium sulfate	25, 50 mg/kg, i.p., 1 h before surgery	↓ ERK phosphorylation, damage, apoptosis, autophagy, inflammation
	Partial Warm I-R I: 60 min-R: 1, 6, 24 h [64]	↑ ERK	5Z-7-oxozeaenol	Adenovirus vectors, i.v., 4 weeks before I-R	No activity changes in ERK ↓ Damage, inflammation
	Partial Warm I-R I: 60 min-R: 1, 4, 24 h [65]	↑ ERK	Methyl-beta-cyclodextrin	10, 25, 50 mg/kg, i.p., 48 and 24 h before I-R	No activity changes in ERK ↑ Damage, apoptosis
	Partial Warm I-R I: 60 min-R: 2, 4, 8, 12, 24 h [66]	↑ ERK	SIRT6 ^-/-^ KO	na	↓ ERK phosphorylation, damage, NF-κB
	Partial Warm I-R I: 60 min-R: 2, 4, 24 h [67]	↑ ERK	Astaxanthin	30, 60 mg/kg, oral, 14 consecutive days before I-R	↓ ERK phosphorylation, damage, apoptosis, ROS, autophagy
	Partial Warm I-R I: 60 min-R: 3, 6, 12, 24 h [68]	↑ ERK	RC-3095	3 mg/kg, i.p., at the time of reperfusion	↓ ERK phosphorylation, damage, inflammation
	Partial Warm I-R I: 60 min-R: 6 h [69]	↑ ERK	Schisantherin A	200 mg/kg/d, oral, 5 days	↓ ERK phosphorylation, damage
	Partial Warm I-R I: 60 min-R: 6 h [70]	↑ ERK	PC	10 min ischemia, 10 min reperfusion	↓ ERK phosphorylation, damage
	Partial Warm I-R I: 90 min-R: 2, 4 h [116]	↑ ERK	SB203580	shRNA plasmids: 10 μg in 2 ml, i.v., 72 h before I-R	↓ ERK phosphorylation, damage
	Partial Warm I-R I: 90 min-R: 3 h [117]	↑ ERK	TBC-1269	10, 20, 40 mg/kg, i.v., 1 h before reperfusion, and 15 min after reperfusion	↓ ERK phosphorylation, damage, neutrophil infiltration, inflammation ↑ Akt, proliferation
	Partial Warm I-R I: 90 min-R: 1, 2, 4, 6 h [97]	↑ ERK	CXCL10 ^-/-^ KO	na	↓ ERK phosphorylation, damage, neutrophil infiltration
Rats	Partial Warm I-R I: 60 min-R: 1, 5 h [98]	↑ ERK	Melatonin	10 mg/kg, i.v., 15 min before ischemia & before reperfusiom	↓ ERK phosphorylation, damage, inflammatory signaling cascades
	Partial Warm I-R I: 60 min-R: 30 min [95]	↑ ERK	IL-1 RA	100-400 µg/100 g, i.p., 30 min before I and immediately after R	↓ ERK phosphorylation, damage, IL-6, TNF-α
	Partial Warm I-R I: 60 min-R: 6 h [80]	↑ ERK	Clotrimazole	50 mg/kg, i.p., 3 days before I-R	↓ Damage, apoptosis ↑ ERK phosphorylation, PXR
	Partial Warm I-R I: 60 min-R: 20, 60, 90 min, 6, 24 h [118]	↑ ERK	FTY720	1 mg/kg, i.v., 20 min before I and 10 min before R	↓ ERK phosphorylation, damage, Egr-1, apoptosis ↑ Cell survival, Akt signaling
	Partial Warm I-R I: 60 min-R: 7 days [84]	↑ ERK	Acanthopanax divaricatus vat albeofructus	600 mg/kg, oral, 2 weeks before I-R	↓ ERK phosphorylation, damage ↑ IL-10
	Partial Warm I-R I: 90 min-R: 1 h [99]	↑ ERK	Cyclopamine	10 mg/kg, i.p., 3 days and 1 h before reperfusion	↓ ERK phosphorylation, damage, inflammation
	Partial Warm I-R I: 60 min-R: 5 days [83]	No activity changes in ERK	PC	15 min ischemia, 15 min reperfusion	↑ ERK phosphorylation
Mice	Total Warm I-R I: 45 min-R: 2, 8, 24 h [86]	↑ ERK	Salidroside	20 mg/kg, i.p., 2 h before I-R	↓ ERK phosphorylation, damage, apoptosis, autophagy
Mice	I-R with Partial (70%) Hepatectomy I: 60 min-R: 24 h [111]	Not evaluated	Sphinganine-1-phosphate	0.1 mg/kg, i.v., before reperfusion and 0.2 mg/kg, s.c., 2 h after reperfusion	↓ Damage, vascular dysfunction ↑ ERK phosphorylation, endothelial cell integrity
	I-R with Partial (30%) Hepatectomy I: 75 min-R: 1 h [100]	↑ ERK	RAGE ^-/-^ KO	na	↓ ERK phosphorylation, damage, neutrophil infiltration
Rats	I-R with Partial (70%) Hepatectomy I: 30 min-R: 3, 9, 18, 21, 24, 30, 48 h [88]	No activity changes in ERK	PC	10 min ischemia, 5 min reperfusion	↓ Damage, apoptosis ↑ ERK phosphorylation, mitochondrial integrity
	I-R with Partial (70%) Hepatectomy I: 60 min-R: 24 h [46]	↑ ERK	TUDCA	100 mg/kg, na, before surgery	↓ Damage, GSK3β activity ↑ ERK phosphorylation
Rats	Liver transplantation Cold I: 1 h-R: 24 h [90]	↑ ERK	Losartan	5 mg/kg, oral, 24 and 1 h before surgery	No activity changes in ERK ↓ Damage ↑ SIRT1
	Liver transplantation Cold I: 80 min-R: 2, 6, 24 h [112]	↑ ERK	Cardiotrophin-1	pAdTrack-CMV vector, na	↓ Damage, Bcl-2, caspase-3, apoptosis ↑ ERK phosphorylation, graft survival
	Liver transplantation Cold I: 6 h-R: 4 h [50]	No activity changes in ERK	Angiotensin II receptor antagonists	AT1R: 5 mg/kg, oral, 24 and 1.5 h before surgery AT2R: 30 mg/kg, i.v., 5 min before surgery	↓ Damage ↑ ERK phosphorylation, survival
	Liver transplantation Cold I: 18 h-R: 1, 3, 6, 24, 48 h [91]	↑ ERK	Carbon monoxide	100 ppm in recipient, 1 h before surgery and 1, 3, and 6 h after surgery	↓ ERK phosphorylation, damage
	Liver transplantation Cold I: 30 h-R: 6, 24 h [113]	↑ ERK	Sotraustaurin	30 mg/kg, oral, 1) 90 min before organ recovery and 3 days in recipients and 2) 3 days in recipients	↓ ERK phosphorylation, damage, caspase-3, apoptosis
Mice	Isolated liver perfused Cold I: 24 h-R: 2 h [114]	No activity changes in ERK	Erythropoietin as additive	10 IU/mL in solution	↓ ERK phosphorylation, damage

Specific ERK Inhibitor: SB203580. Non Selective ERK Modulators: 5Z-7-oxozeaenol: TAK1 inhibitor; Acanthopanax divaricatus vat. Albeofructus; Angiotensin II receptor antagonists; Astaxanthin; Carbon monoxide; Cardiotrophin-1; Clotrimazole; CXCL10; Cyclopamine; Erythropoietin; FTY720; IL-1 RA receptor antagonist; Losartan; Melatonin; Methyl-beta-cyclodextrin: Caveolae disruptor; PC; Propylene glycol alginate sodium sulfate; RAGE; RC-3095; Remifentanil; Salidroside; Schisantherin A; SIRT6; Sotraustaurin; Sphinganine-1-phosphate; TBC-1269: selectin inhibitor; TUDCA.

**Table 7:** Effect of strategies that regulate MAPK in human hepatic ischemia- reperfusion.

Experimental Model			Changes in MAPK Induced by I-R	Treatment with MAPK Modulators	Effect of MAPK Modulators
Species	Surgical Procedure	Type of Liver			
**Human**	Isolated Liver Perfused Cold I: >10 h SCS + 4h HMP R: 2 h [119]	Marginal (steatotic, not usable DCD, donor malignancy, failed allocation, atheromatosis, poor perfusion, rising liver function test)	↑ MAPK	Hypothermic machine perfusion	No benefits on histological score. ↓ MAPK, AST, LDH

SCS: Simple cold storage; HMP: Hypothermic machine perfusion.

## Abbreviations

A2-R	Adenosine 2 receptor
ACH	Acetylcholine
AGE	Advanced glycation endproduct
LD	Linear dichroism
Akt	Protein kinase B
AMP	AMP Adenosine monophosphate
Ang	Angiotensin
ANP	Atrial natriuretic peptide
AP-1	Activating protein
ASK1	Apoptosis signal-regulating kinase 1
AST	Aspartate aminotransferase
ATF-2	Activating transcription factor 2
ATP	Adenosine triphosphate
Bcl-2	B-cell lymphoma 2
cAMP	Cyclic adenosine monophosphate
cGMP	Guanosine 3’,5’-cyclic monophosphate
CO	Carbon monoxide
CS-1	Connecting segment-1 peptide
CXCL10	C-X-C motif chemokine ligand 10
DCD	Donation after circulatory death
EGF	Epidermal growth factor
eNOS	Endothelial nitric oxide synthase
ER	Endoplasmic reticulum
ERK 1/2	Extracellular signal-regulated protein kinases
ET-1	Endothelin-1
GM-CSF	Granulocyte-macrophage colony-stimulating factor
GSH	Glutathione
HFD	High fat diet
HMP	Hypothermic machine perfusion
HO-1	Heme oxygenase-1
HSF-1	Heat shock transcription factor-1
HSP	Heat shock protein
I-R	Ischemia-reperfusion
ICAM	Intracellular cell adhesion molecule
IGF	Insulin-like growth factor
IL	Interleukin
iNOS	Inducible nitric oxide synthase
i.p	Intraperitoneal
i.v	Intravenous
JNK	c-Jun N-terminal kinase
KO	Knockout
LDH	Lactate dehydrogenase
LTB4	Leucotriene B4
MAPKs	Mitogen-activated protein kinases
MEF2c	Myocyte enhancer factor-2
Min	Minutes
NADPH	Nicotinamide adenine dinucleotide phosphate
NAFLD	Non-alcoholic fatty liver disease
NASH	Non-alcoholic steatohepatitis
NDQI-1	NQDI-1–2,7-Dihydro-2,7-dioxo-3H-naphth-o[1,2,3dequin-oline-1-carboxylic acid ethyl ester]
NF-κB	Nuclear factor κ Beta
NO	Nitric oxide
PAF	Platelet activating factor
PC	Ischemic preconditioning
PDGF	Platelet-derived growth factor
PI3K	Phosphoinositide 3-kinase
PKC	Protein kinase C
PLC	Phospholipase C
RAGE	Receptor advanced glycation endproduct
RBP4	Retinol binding protein 4
ROS	Reactive oxygen species
SCS	Simple cold storage
SIRT1	Sirtuin-1
SLPI	Secretory leukocyte protease inhibitor
SOD	Superoxide dismutase
STAT3	Signal transducer and activator of transcription-3
Thr	Threonine
TNF-α	Tumor necrosis factor alpha
TUDCA	Tauroursodeoxycholic acid
Tyr	Tyrosine
X/XOD	Xanthine/xanthine oxidase
Z-Asp-cmk	Z-Asp-2,6-dichorobenzoyloxymethylketone

## References

[1] Casillas-Ramírez A., Mosbah I.B., Ramalho F., Roselló-Catafau J., Peralta C. (2006). Past and future approaches to ischemia-reperfusion lesion associated with liver transplantation. Life Sci..

[2] Serafin A., Rosello-Catafau J., Prats N., Xaus C., Gelpi E., Peralta C. (2002). Ischemic preconditioning increases the tolerance of fatty liver to hepatic ischemia-reperfusion injury in the rat. Am. J. Pathol..

[3] Clavien P., Harvey P., Strasberg S. (1992). Preservation and reperfusion injuries in liver allografts. An overview and synthesis of current studies. Transplantation.

[4] Huguet C., Gavelli A., Chieco P., Bona S., Harb J., Joseph J.M., Jobard J., Gramaglia M., Lasserre M. (1992). Liver ischemia for hepatic resection: Where is the limit?. Surgery.

[5] Busuttil R.W., Tanaka K. (2003). The utility of marginal donors in liver transplantation. Liver Transpl..

[6] Samuel V.T., Shulman G.I. (2018). Nonalcoholic fatty liver disease as a nexus of metabolic and hepatic diseases. Cell Metab..

[7] Pratap A., Panakanti R., Yang N., Lakshmi R., Modanlou K.A., Eason J.D., Mahato R.I. (2011). Cyclopamine attenuates acute warm ischemia reperfusion injury in cholestatic rat liver: Hope for marginal livers. Mol. Pharm..

[8] Nadig S.N., Periyasamy B., Shafizadeh S.F., Polito C., Fiorini R.N., Rodwell D., Evans Z., Cheng G., Dunkelberger D., Schmidt M. (2004). Hepatocellular ultrastructure after ischemia/reperfusion injury in human orthotopic liver transplantation. J. Gastrointest. Surg..

[9] Verran D., Kusyk T., Painter D., Fisher J., Koorey D., Strasser S., Stewart G., McCaughan G. (2003). Clinical experience gained from the use of 120 steatotic donor livers for orthotopic liver transplantation. Liver Transpl..

[10] Schaubel D.E., Sima C.S., Goodrich N.P., Feng S., Merion R.M. (2008). The survival benefit of deceased donor liver transplantation as a function of candidate disease severity and donor quality. Am. J. Transplant..

[11] Ploeg R.J., D’ Alessandro A.M., Knechtle S.J., Stegall M.D., Pirsch J.D., Hoffmann R.M., Sasaki T., Sollinger H.W., Belzer F.O., Kalayoglu M. (1993). Risk factors for primary dysfunction after liver transplantation-a multivariate analysis. Transplantation.

[12] Behrns K.E., Tsiotos G.G., DeSouza N.F., Krishna M.K., Ludwig J., Nagorney D.M. (1998). Hepatic steatosis as a potential risk factor for major hepatic resection. J. Gastrointest. Surg..

[13] Lopez-Neblina F., Toledo A.H., Toledo-Peryra L.H. (2005). Molecular biology of apoptosis in ischemia and reperfusion. J. Investig. Surg..

[14] King L.A., Toledo A.H., Rivera-Chavez F.A., Toledo-Pereyra L.H. (2009). Role of p38 and JNK in liver ischemia and reperfusion. J. Hepatobiliary Pancreat. Surg..

[15] Kobayashi M., Takeyoshi I., Yoshinari D., Matsumoto K., Morishita Y. (2006). The role of mitogen-activated protein kinases and the participation of intestinal congestion in total hepatic ischemia–reperfusion injury. Hepatogastroenterology.

[16] Kumar S., Boehm J., Lee J.C. (2003). p38 MAP Kinases: Key signalling molecules as therapeutic targets for inflammatory diseases. Nat. Rev. Drug Discov..

[17] Schnabl B., Bradham C.A., Bennett B.L., Manning A.M., Stefanovic B., Brenner D.A. (2001). TAK1/JNK and p38 have opposite effects on rat hepatic stellate cells. Hepatology.

[18] Toledo-Pereyra L.H., Toledo A.H., Walsh J., Lopez-Neblina F. (2004). Molecular signaling pathways in ischemia/reperfusion. Exp. Clin. Transplant..

[19] Bradham C.A., Stachlewitz R.F., Gao W., Qian T., Jayadev S., Jenkins G., Hannun Y., Lemasters J.J., Thurman R.G., Brenner D.A. (1997). Reperfusion after liver transplantation in rats differentially activates the mitogen-activated protein kinases. Hepatology.

[20] Ding C., Wilding J.P., Bing C. (2013). 1,25-dihydroxyvitamin D3 protects against macrophage-induced activation of NFκB and MAPK signaling and chemokine release in human adipocytes. PLoS ONE.

[21] Mutt S.J., Karhu T., Lehtonen S., Carlberg C., Saarnio J., Sebert S., Hypponen E., Jarvelin M.R., Herzing K.H. (2012). Inhibition of cytokine secretion from adipocytes by 1,25-dihydroxyvitamin D via the NF-κB pathway. FASEB J..

[22] Meeker S., Seamons A., Paik J., Treuting P.M., Brabb T., Grady W.M., Maggio-prince L. (2014). Increased dietary vitamin D suppresses MAPK signaling, colitis, and colon cancer. Cancer Res..

[23] Nelson J.E., Roth C.L., Wilson L.A., Yates K.P., Aouizerat B., Morgan-Stevenson V., Whalen E., Hoofnagle A., Mason M., Gersuk V. (2016). Vitamin D deficiency is associated with increased risk of non-alcoholic steatohepatitis in adults with non-alcoholic fatty liver disease: Possible role for MAPK and NF-κB?. Am. J. Gastroenterol..

[24] Vega M.I., Huerta-Yepaz S., Garban H., Jazirehi A., Emmanouilides C., Bonavida B. (2004). Rituximab inhibits p38 MAPK activity in 2F7 B NHL and decreases IL-10 transcription: Pivotal role of p38 MAPK in drug resistance. Oncogene.

[25] Hu X., Chen J., Wang L., Ivashkiv L.B. (2007). Crosstalk among Jak-STAT, Toll-like receptor, and ITAM-dependent pathways in macrophage activation. J. Leukocyte Biol..

[26] Younossi Z.M., Karrar A., Pierobon M., Birerdinc A., Stepanova M., Abdelatif D., Younoszai Z., Jeffers T., Felix S., Jeiran K. (2018). An exploratory study examining how nano-liquid chromatography-mass spectrometry and phosphoproteomics can differentiate patients with advanced fibrosis and higher percentage collagen in non-alcoholic fatty liver disease. BMC Med..

[27] Wu Q., Li S., Li X., Sui Y., Yang Y., Dong L., Xie B., Sun Z. (2015). Inhibition of advanced glycation end product formation by lotus seedpod oligomeric procyanidins through RAGE-MAPK signaling and NF-κB activation in high-fat-diet rats. J. Agric. Food Chem..

[28] Banga N.R., Homer-Vanniasinkam S., Graham A., Al-Mukhtar A., White S.A., Prasad K.R. (2005). Ischaemic preconditioning in transplantation and major resection of the liver. Br. J. Surg..

[29] Fan C., Zwacka R.M., Engelhardt J.F. (1999). Therapeutic approaches for ischemia/reperfusion injury in the liver. J. Mol. Med..

[30] Jaeschke H. (2003). Molecular mechanisms of hepatic ischemia-reperfusion injury and preconditioning. Am. J. Physiol. Gastrointest. Liver Physiol..

[31] Lentsch A.B., Kato A., Yoshidome H., McMasters K.M., Edwards M.J. (2000). Inflammatory mechanisms and therapeutic strategies for warm hepatic ischemia/reperfusion injury. Hepatology.

[32] Massip-Salcedo M., Roselló-Catafau J., Prieto J., Avíla M.A., Peralta C. (2007). The response of the hepatocyte to ischemia. Liver Int..

[33] Kobayashi M., Takeyoshi I., Yoshinari D., Matsumoto K., Morishita Y. (2002). P38 mitogen-activated protein kinase inhibition attenuates ischemia–reperfusion injury of the rat liver. Surgery.

[34] Liang R., Nickkholgh A., Hoffmann K., Kern M., Schneider H., Sobirey M., Zorn M., Buchler M.W., Schemmer P. (2009). Melatonin protects from hepatic reperfusion injury through inhibition of IKK and JNK pathways and modification of cell proliferation. J. Pineal Res..

[35] Toledo-Pereyra L.H., Lopez-Neblina F., Toledo A.H. (2008). Protein kinases in organ ischemia and reperfusion. J. Investig. Surg..

[36] Tsung A., Hoffman R.A., Izuishi K., Critchlow N.D., Nakao A., Chan M.H., Lotze M.T., Geller D.A., Billiar T.R. (2005). Hepatic ischemia/reperfusion injury involves functional TLR4 signaling in nonparenchymal cells. J. Immunol..

[37] Schwabe R.F., Brenner D.A. (2006). Mechanisms of liver injury. I. TNF-α induced liver injury: Role of IKK, JNK, and ROS pathways. Am. J. Physiol. Gastrointest. Liver Physiol..

[38] Onishi I., Shimizu K., Tani T., Hashimoto T., Miwa K. (1999). JNK activation and apoptosis during ischemia–reperfusion. Transplant. Proc..

[39] Yoshinari D., Takeyoshi I., Kobayashi M., Koyama T., Iijima K., Ohwada S., Matsumoto K., Morishita Y. (2001). Effects of a p38 mitogen-activated protein kinase inhibitor as an additive to university of wisconsin solution on reperfusion injury in liver transplantation. Transplantation.

[40] Massip-Salcedo M., Casillas-Ramirez A., Franco-Gou R., Bartrons R., Ben Mosbah I., Serafin A., Rosello-Catafau J., Peralta C. (2006). Heat shock proteins and mitogen-activated protein kinases in steatotic livers undergoing ischemia–reperfusion: Some answers. Am. J. Pathol..

[41] Rosseland C., Wierød L., Oksvold M.P., Werner H., Østvold A.C., Thoresen G.H., Paulsen R.E., Huitfeldt H.S., Skarpen E. (2005). Cytoplasmic retention of peroxide-activated ERK provides survival in primary cultures of rat hepatocytes. Hepatology.

[42] Widmann C., Gibson S., Jarpe M.B., Johnson G.L. (1999). Mitogen-activated protein kinase: Conservation of a three-kinase module from yeast to human. Physiol. Rev..

[43] Kaminska B. (2005). MAPK signaling pathways as molecular targets for anti-inflammatory therapy-from molecular mechanisms to therapeutic benefits. Biochim. Biophys. Acta.

[44] Casillas-Ramírez A., Zaouali A., Padrissa-Altés S., Ben Mosbah I., Pertosa A., Alfany-Fernández I., Bintanel-Morcillo M., Xaus C., Rimola A., Rodes J., Rosello-Catafau J., Peralta C. (2009). Insulin-like growth factor and epidermal growth factor treatment: New approaches to protecting steatotic livers against ischemia-reperfusion injury. Endocrinology.

[45] Ramalho F.S., Alfany-Fernandez I., Casillas-Ramirez A., Massip-Salcedo M., Serafín A., Rimola A., Arroyo V., Rodes J., Rosello-Catafau J., Peralta C. (2009). Are angiotensin II receptor antagonists useful strategies in steatotic and nonsteatotic livers in conditions of partial hepatectomy under ischemia-reperfusion?. J. Pharmacol. Exp. Ther..

[46] Ben Mosbah I., Alfany-Fernandez I., Martel C., Zaouali M.A., Bintanel-Morcillo M., Rimola A., Rodes J., Brenner C., Rosello-Catafau J., Peralta C. (2010). Endoplasmic reticulum stress inhibition protects steatotic and non-steatotic livers in partial hepatectomy under ischemia-reperfusion. Cell Death Dis..

[47] Cursio R., Filippa N., Miele C., Van Obberghen E., Gugenheim J. (2006). Involvement of protein kinase B and mitogen-activated protein kinases in experimental normothermic liver ischemia-reperfusion injury. Br. J. Surg..

[48] Massip-Salcedo M., Zaouali M.A., Padrissa-Altés S., Casillas-Ramirez A., Rodés J., Roselló-Catafau J., Peralta C. (2008). Activation of peroxisome proliferator-activated receptor-alpha inhibits the injurious effects of adiponectin in rat steatotic liver undergoing ischemia-reperfusion. Hepatology.

[49] Fernández L., Carrasco-Chaumel E., Serafín A., Xaus C., Grande L., Rimola A., Roselló-Catafau J., Peralta C. (2004). Is ischemic preconditioning a useful strategy in steatotic liver transplantation?. Am. J. Transplant..

[50] Alfany-Fernández I., Casillas-Ramírez A., Bintanel-Morcillo M., Brosnihan K.B., Ferrario C.M., Serafin A., Rimola A., Rodés J., Rosellò-Catafau J., Peralta C. (2009). Therapeutic targets in liver transplantation: Angiotensin II in nonsteatotic grafts and angiotensin-(1-7) in steatotic grafts. Am. J. Transplant..

[51] Keller M., Gerbes A.L., Kulhanek-Heinze S., Gerwig T., Grutzner U., van Rooijen N., Vollmar A.M., Kiemer A.K. (2005). Hepatocyte cytoskeleton during ischemia and reperfusion-influence of ANP-mediated p38 MAPK activation. World J. Gastroenterol..

[52] Li S., Takahara T., Fujino M., Fukuhara Y., Sugiyama T., Li X.K., Takahara S. (2017). Astaxanthin prevents ischemia-reperfusion injury of the steatotic liver in mice. PLoS ONE.

[53] Liang T., Xu S., Yu J., Shen K., Li D., Zheng S. (2005). Activation pattern of mitogen-activated protein kinases in early phase of different size liver isografts in rats. Liver Transpl..

[54] Ping P., Zhang J., Huang S., Cao X., Tang X.L., Li R.C., Zheng Y.T., Qiu Y., Clerk A., Sugden P. (1999). PKC-dependent activation of p46/p54 JNKs during ischemic preconditioning in conscious rabbits. Am. J. Physiol..

[55] Guo X., Gerl R.E., Schrader J.W. (2003). Defining the involvement of p38α MAPK in the production of anti and proinflammatory cytokines using an SB203580-resistant form of the kinase. J. Biol. Chem..

[56] Dumeus S., Shibu M.A., Lin W.T., Wang M.F., Lai C.H., Shen C.Y., Lin Y.M., Viswanadha V.P., Kuo W.W., Huang C.Y. (2018). Bioactive peptide improves diet-induced hepatic fat deposition and hepatocyte proinflammatory response in SAMP8 ageing mice. Cell Physiol. Biochem..

[57] Karin M. (2004). Mitogen activated protein kinases as targets for development of novel anti-inflammatory drugs. Ann. Rheum. Dis..

[58] Kobayashi K., Oshima K., Muraoka M., Akao T., Totsuka O., Shimizu H., Sato H., Tanaka K., Konno K., Matsumoto K. (2007). Effect of atrial natriuretic peptide on ischemia–reperfusion injury in a porcine total hepatic vascular exclusion model. World J. Gastroenterol..

[59] Imarisio C., Alchera E., Revanna C.B., Valente G., Follenzi A., Trisolini E., Boldorini R., Carini R. (2017). Oxidative and ER stress-dependent ASK1 activation in steatotic hepatocytes and Kupffer cells sensitizes mice fatty liver to ischemia/reperfusion injury. Free Radic. Biol. Med..

[60] Deng J., Feng J., Liu T., Lu X., Wang W., Liu N., Lv Y., Liu Q., Guo C., Zhou Y. (2018). Beraprost sodium preconditioning prevents inflammation, apoptosis, and autophagy during hepatic ischemia-reperfusion injury in mice via the P38 and JNK pathways. Drug Des. Dev. Ther..

[61] Xu S., Niu P., Chen K., Xia Y., Yu Q., Liu N., Li J., Li S., Wu L., Feng J. (2017). The liver protection of propylene glycol alginate sodium sulfate preconditioning against ischemia reperfusion injury: Focusing MAPK pathway activity. Sci. Rep..

[62] Yang L., Wang W., Wang X., Zhao J., Xiao L., Gui W., Fan H., Xia J., Li Z., Yan J. (2019). Creg in hepatocytes ameliorates liver ischemia/reperfusion injury in a TAK1-dependent manner in mice. Hepatology.

[63] Ocuin L.M., Zeng S., Cavnar M.J., Sorenson E.C., Bamboat Z.M., Greer J.B., Kim T.S., Popow R., DeMatteo R.P. (2012). Nilotinib protects the murine liver from ischemia/reperfusion injury. J. Hepatol..

[64] Wang X., Mao W., Fang C., Tian S., Zhu X., Yang L., Huang Z., Li H. (2018). Dusp14 protects against hepatic ischaemia-reperfusion injury via Tak1 suppression. J. Hepatol..

[65] Kang J.W., Lee S.M. (2014). Impaired expression of caveolin-1 contributes to hepatic ischemia and reperfusion injury. Biochem. Biophys. Res. Commun..

[66] Zhang S., Jiang S., Wang H., Di W., Deng C., Jin Z., Yi W., Xiao X., Nie Y., Yang Y. (2018). SIRT6 protects against hepatic ischemia/reperfusion injury by inhibiting apoptosis and autophagy related cell death. Free Radic. Biol. Med..

[67] Li J., Wang F., Xia Y., Dai W., Chen K., Li S., Liu T., Zheng Y., Wang J., Lu W. (2015). Astaxanthin pretreatment attenuates hepatic ischemia reperfusion-induced apoptosis and autophagy via the ROS/MAPK pathway in mice. Mar. Drugs.

[68] Guo L., Wu X., Zhang Y., Wang F., Li J., Zhu J. (2019). Protective effects of gastrin-releasing peptide receptor antagonist RC-3095 in an animal model of hepatic ischemia/reperfusion injury. Hepatol. Res..

[69] Zheng N., Liu F., Lu H., Zhan Y., Zhang M., Guo W., Ding G. (2017). Schisantherin A protects against liver ischemia-reperfusion injury via inhibition of mitogen-activated protein kinase pathway. Int. Immunopharmacol..

[70] Shin J.K., Kang J.W., Lee S.M. (2016). Enhanced nitric oxide-mediated autophagy contributes to the hepatoprotective effects of ischemic preconditioning during ischemia and reperfusion. Nitric Oxide.

[71] Sun L., Wu Q., Nie Y., Cheng N., Wang R., Wang G., Zhang D., He H., Ye R.D., Qian F. (2018). A role for MK2 in enhancing neutrophil-derived ROS production and aggravating liver ischemia/reperfusion injury. Front. Immunol..

[72] Teoh N., Dela Pena A., Farrell G. (2002). Hepatic ischemic preconditioning in mice is associated with activation of NF-κB, p38 kinase, and cell cycle entry. Hepatology.

[73] Sakai N., Van Sweringen H.L., Quillin R.C., Schuster R., Blanchard J., Burns J.M., Tevar A.D., Edwards M.J., Lentsch A.B. (2012). Interleukin-33 is hepatoprotective during liver ischemia/reperfusion in mice. Hepatology.

[74] Teoh N., Laclercq I., Pena A.D., Farrell G. (2003). Low dose TNF-α protects against hepatic ischemia–reperfusion injury in mice: Implications for preconditioning. Hepatology.

[75] Teoh N., Field J., Sutton J., Farrell G. (2004). Dual role of tumor necrosis factor-α in hepatic ischemia-reperfusion injury: Studies in tumor necrosis factor-α gene knockout mice. Hepatology.

[76] Zhou L., Zhao D., An H., Zhang H., Jiang C., Yang B. (2015). Melatonin prevents lung injury induced by hepatic ischemia-reperfusion through anti-inflammatory and anti-apoptosis effects. Int. Immunopharmacol..

[77] Gendy A.M., Abdallah D.M., El-Abhar H.S. (2017). The potential curative effect of rebamipide in hepatic ischemia/reperfusion injury. Naunyn Schmiedebergs Arch. Pharmacol..

[78] Yamaguchi K., Kawahara T., Kumakura S., Hua J., Kugimiya T., Nagaoka I., Inada E. (2010). Effect of oprinome, a phosphodiesterase III inhibitor, on hepatic ischemia-reperfusion injury in rats. Shock.

[79] Sun P.P., Yuan F., Xu J., Sai K., Chen J., Guan S. (2014). Cryptotanshinone ameliorates hepatic normothermic ischemia and reperfusion injury in rats by anti-mitochondrial apoptosis. Biol. Pharm. Bull..

[80] Iannelli A., de Sousa G., Zucchini N., Saint-Paul M.C., Gugenheim J., Rahmani R. (2001). Anti-apoptotic pro-survival effect of clotrimazole in a normothermic ischemia reperfusion injury animal model. J. Surg. Res..

[81] Rao J., Zhang C., Wang P., Lu L., Zhang F. (2010). All-trans retinoic acid alleviates hepatic ischemia/reperfusion injury by enhancing manganese superoxide dismutase in rats. Biol. Pharm. Bull..

[82] Schauer R.J., Gerbes A.L., Vonier D., op den Winkel M., Fraunberger P., Bilzer M. (2003). Induction of cellular resistance against Kupffer cell-derived oxidant stress: A novel concept of hepatoprotection by ischemic preconditioning. Hepatology.

[83] Kapoor S., Berishvili E., Bandi S., Gupta S. (2014). Ischemic preconditioning affects long-term cell fate through DNA damage-related molecular signaling and altered proliferation. Am. J. Pathol..

[84] Lim E.J., Do G.M., Shin J.H., Kwon O. (2013). Protective effects of Acanthopanax divaricatus vat. albeofructus and its active compound on ischemia-reperfusion injury of rat liver. Biochem. Biophys. Res. Commun..

[85] Ruess D.A., Probst M., Marjanovic G., Wittel U.A., Hopt U.T., Keck T., Bausch D. (2016). HDACi valproic acid (VPA) and suberoylanilide hydroxamic acid (SAHA) delay but fail to protect against warm hepatic ischemia-reperfusion injury. PLoS ONE.

[86] Feng J., Zhang Q., Mo W., Wu L., Li S., Li J., Liu T., Xu S., Fan X., Guo C. (2017). Salidroside pretreatment attenuates apoptosis and autophagy during hepatic ischemia-reperfusion injury by inhibiting the mitogen-activated protein kinase pathway in mice. Drug Des. Dev. Ther..

[87] Kawai K., Yokoyama Y., Kokuryo T., Watanabe K., Kitagawa T., Nagino M. (2010). Inchinkoto, an herbal medicine, exerts beneficial effects in the rat liver under stress with hepatic ischemia-reperfusion and subsequent hepatectomy. Ann. Surg..

[88] Ben Mosbah I., Duval H., Mbatchi S.F., Ribault C., Grandadam S., Pajaud J., Morel F., Boudjema K., Compagnon P., Corlu A. (2014). Intermittent selective clamping improves rat liver regeneration by attenuating oxidative and endoplasmic reticulum stress. Cell Death Dis..

[89] Tang Y., Zhang W., Zhang Y., Wang W., Yao F., Yan J., Wan C. (2014). 5′-Methylthioadenosine attenuates ischemia reperfusion injury after liver transplantation in rats. Inflammation.

[90] Pantazi E., Bejaoui M., Zaouali M.A., Folch-Puy E., Pinto-Rolo A., Panisello A., Palmeira C.M., Roselló-Catafau J. (2015). Losartan activates sirtuin 1 in rat reduced-size orthotopic liver transplantation. World J. Gastroenterol..

[91] Kaizu T., Ikeda A., Nakao A., Tsung A., Toyokawa H., Ueki S., Geller D.A., Murase N. (2008). Protection of transplant-induced hepatic ischemia/reperfusion injury with carbon monoxide via MEK/ERK1/2 pathway downregulation. Am. J. Physiol. Gastrointest. Liver Physiol..

[92] Duarte S., Shen X.D., Fondevila C., Busuttil R.W., Coito A.J. (2012). Fibronectin-α4β1 interactions in hepatic cold ischemia and reperfusion injury: Regulation of MMP-9 and MT1-MMP via the p38 MAPK pathway. Am. J. Transplant..

[93] Kiemer A.K., Kulhanek-Heinze S., Gerwig T., Gerbes A.L., Vollmar A.M. (2002). Stimulation of p38 MAPK by hormonal preconditioning with atrial natriuretic peptide. World J. Gastroenterol..

[94] Amersi F., Shen X.D., Anselmo D., Melinek J., Iyer S., Southard D.J., Katori M., Volk H.D., Busuttil R.W., Buelow R. (2002). Ex vivo exposure to carbon monoxide prevents hepatic ischemia/reperfusion injury through p38 MAP kinase pathway. Hepatology.

[95] Bendinelli P., Piccoletti R., Maroni P., Bernelli-Zazzera A. (1996). The MAP kinase cascades are activated during post-ischemic liver reperfusion. FEBS Lett..

[96] Lee K.H., Kim S.E., Lee Y.S. (2006). SP600125, a selective JNK inhibitor, aggravates hepatic ischemia-reperfusion injury. Exp. Mol. Med..

[97] Zhai Y., Shen X.D., Gao F., Zhao A., Freitas M.C., Lassman C., Luster A.D., Busuttil R.W., Kupiec-Weglinski J.W. (2008). CXCL10 regulates liver innate immune response against ischemia and reperfusion injury. Hepatology.

[98] Kang J.W., Koh E.J., Lee S.M. (2011). Melatonin protects liver against ischemia and reperfusion injury through inhibition of toll-like receptor signaling pathway. J. Pineal Res..

[99] Pratap A., Panakanti R., Yang N., Eason J.D., Mahato R.I. (2010). Inhibition of endogenous hedgehog signaling protects against acute liver injury after ischemia reperfusion. Pharm. Res..

[100] Zeng S., Dun H., Ippagunta N., Rosario R., Zhang Q.Y., Lefkowitch J., Yan S.F., Schmidt A.M., Emond J.C. (2009). Receptor for advanced glycation end product (RAGE)-dependent modulation of early growth response-1 in hepatic ischemia/reperfusion injury. J. Hepatol..

[101] Vilatoba M., Eckstein C., Bilbao G., Frennete L., Eckhoff D.E., Contreras J.L. (2005). 17β-estradiol differentially activates mitogen-activated protein-kinases and improves survival following reperfusion injury of reduced-size liver in mice. Transplant. Proc..

[102] Uehara T., Peng X.X., Bennett B., Satoh Y., Friedman G., Currin R., Brenner D.A., Lemasters J. (2004). c-Jun N-terminal kinase mediates hepatic injury after rat liver transplantation. Transplantation.

[103] Uehara T., Bennett B., Sakata S.T., Satoh Y., Bilter G.K., Westwick J.K., Brenner D.A. (2005). JNK mediates hepatic ischemia reperfusion injury. J. Hepatol..

[104] Serafín A., Roselló-Catafau J., Prats N., Gelpí E., Rodés J., Peralta C. (2004). Ischemic preconditioning affects interleukin release in fatty livers of rats undergoing ischemia/reperfusion. Hepatology.

[105] Westwick J.K., Weitzel C., Leffert H.L., Brenner D.A. (1995). Activation of Jun kinase is an early event in hepatic regeneration. J. Clin. Investig..

[106] Rai R.M., Yang S.Q., McClain C., Karp C.L., Klein A.S., Diehl A.M. (1996). Kupffer cell depletion by gadolinium chloride enhances liver regeneration after partial hepatectomy in rats. Am. J. Physiol..

[107] Suzuki S., Nakamura S., Sakaguchi T., Ochiai H., Konno H., Baba S., Baba S. (1997). Alteration of reticuloendothelial phagocytic function and tumor necrosis factor-alpha production after total hepatic ischemia. Transplantation.

[108] Watanabe M., Chijiiwa K., Kameoka N., Yamaguchi K., Kuroki S., Tanaka M. (2000). Gadolinium pretreatment decreases survival and impairs liver regeneration after partial hepatectomy under ischemia/reperfusion in rats. Surgery.

[109] Yang Y., Chen C., Cui C., Jiao Y., Li P., Zhu L., Yu W., Xia Q., Wen D., Yang L. (2019). Indispensable role of β-arrestin2 in the protection of remifentanil preconditioning against hepatic ischemic reperfusion injury. Sci. Rep..

[110] Cobb M.H. (1999). MAP kinase pathways. Prog. Biophys. Mol. Biol..

[111] Park S.W., Kim M., Chen S.W., Brown K.M., D’Agati V.D., Lee H.T. (2010). Sphinganine-1-phosphate protects kidney and liver after hepatic ischemia and reperfusion in mice through S1P1 receptor activation. Lab. Investig..

[112] Song J., Zhang Y.W., Yao A.H., Yu Y., Hua Z.Y., Pu L.Y., Li G.Q., Li X.C., Zhang F., Sheng G.Q. (2008). Adenoviral cardiotrophin-1 transfer improves survival and early graft function after ischemia and reperfusion in rat small-for-size liver transplantation model. Transpl. Int..

[113] Kamo N., Shen X.D., Ke B., Busuttil R.W., Kupiec-Weglinski J.W. (2011). Sotrastaurin, a protein kinase C inhibitor, ameliorates ischemia and reperfusion injury in rat orthotopic liver transplantation. Am. J. Transplant..

[114] Eipel C., Hübschmann U., Abshagen K., Wagner K.F., Menger M.D., Vollmar B. (2010). Erythropoietin as additive of HTK preservation solution in cold ischemia/reperfusion injury of steatotic livers. J. Surg. Res..

[115] Moore C., Shen X.D., Fondevila C., Coito A.J. (2005). Fibronectin-alpha4beta1 integrin interactions modulate p42/44 MAPK phosphorylation in steatotic liver cold ischemia-reperfusion injury. Transplant. Proc..

[116] Xu D., Zhu J., Jeong S., Li D., Hua X., Huang L., Zhang J., Luo Y., Xia Q. (2018). Rictor deficiency aggravates hepatic ischemia/reperfusion injury in mice by suppressing autophagy and regulating MAPK signaling. Cell Physiol. Biochem..

[117] Toledo-Pereyra L.H., Lopez-Neblina F., Reuben J.S., Toledo A.H., Ward P.A. (2004). Selectin inhibition modulates Akt/MAPK signaling and chemokine expression after liver ischemia-reperfusion. J. Investig. Surg..

[118] Man K., Ng K.T., Lee T.K., Lo C.M., Sun C.K., Li X.L., Zhao Y., Ho J.W., Fan S.T. (2005). FTY720 attenuates hepatic ischemia-reperfusion injury in normal and cirrhotic livers. Am. J. Transplant..

[119] Vekemans K., van Pelt J., Komuta M., Wylin T., Heedfeld V., Detry O., Monbaliu D., Pirenne J. (2011). Attempt to rescue discarded human liver grafts by end ischemic hypothermic oxygenated machine perfusion. Transplant. Proc..

[120] Murry C.E., Jennings R.B., Reimer K.A. (1986). Preconditioning with ischemia: A delay of lethal cell injury in ischemic myocardium. Circulation.

[121] Przyklenk K., Bauer B., Ovize M., Kloner R.A., Whittaker P. (1993). Regional ischemic “preconditioning” protects remote virgin myocardium from subsequent sustained coronary occlusion. Circulation.

[122] Bahde R., Spiegel H.U. (2010). Hepatic ischaemia-reperfusion injury from bench to bedside. Br. J. Surg..

[123] Desai K.K., Dikdan G.S., Shareef A., Koneru B. (2008). Ischemic preconditioning of the liver: A few perspectives from the bench to bedside translation. Liver Transpl..

[124] Clavien P.A., Selzner M., Rudiger H.A., Graft R., Kadry Z., Rousson V., Jochum W. (2003). A prospective randomized study in 100 consecutive patients undergoing major liver resection with versus without ischemic preconditioning. Ann. Surg..

[125] Koneru B., Shareef A., Dikdan G., Desai K., Klein K.M., Peng B., Wachsberg R.H., de la Torre A.N., Debroy M., Fisher A., Wilson D.J., Samanta A.K. (2007). The ischemic preconditioning paradox in deceased donor liver transplantation-evidence from a prospective randomized single blind clinical trial. Am. J. Transplant..

[126] Robertson F.P., Magill L.J., Wright G.P., Fuller B., Davidson B.R. (2016). A systematic review and meta-analysis of donor ischaemic preconditioning in liver transplantation. Transpl. Int..

[127] Jiménez-Castro M.B., Meroño N., Mendes-Braz M., Gracia-Sancho J., Martínez-Carreres L., Cornide-Petronio M.E., Casillas-Ramirez A., Rodés J., Peralta C. (2015). The effect of brain death in rat steatotic and non-steatotic liver transplantation with previous ischemic preconditioning. J. Hepatol..

[128] Cornide-Petronio M.E., Negrete-Sánchez E., Mendes-Braz M., Casillas-Ramírez A., Bujaldon E., Meroño N., Martínez-Carreres L., Gracia-Sancho J., Rodés J., Jiménez-Castro M.B. (2016). The effect of high-mobility group box 1 in rat steatotic and nonsteatotic liver transplantation from donors after brain death. Am. J. Transplant..

[129] Franco-Gou R., Peralta C., Massip-Salcedo M., Xaus C., Serafín A., Roselló-Catafau J. (2004). Protection of reduced-size liver for transplantation. Am. J. Transplant..

[130] Barrier A., Olaya N., Chiappini F., Roser F., Scatton O., Artus C., Franc B., Dudoit S., Flahault A., Debuire B. (2005). Ischemic preconditioning modulates the expression of several genes, leading to the overproduction of IL-1Ra, iNOS, and Bcl-2 in a human model of liver ischemia-reperfusion. FASEB J..

[131] Cutrin J.C., Perrelli M.G., Cavalieri B., Peralta C., Rosello-Catafau J., Poli G. (2002). Microvascular dysfunction induced by reperfusion injury and protective effect of ischemic preconditioning. Free Radic. Biol. Med..

[132] Serafin A., Fernandez-Zabalegui L., Prats N., Wu Z.Y., Rosello-Catafau J., Peralta C. (2004). Ischemic preconditioning: Tolerance to hepatic ischemia-reperfusion injury. Histol. Histopathol..

[133] Carini R., Albano E. (2003). Recent insights on the mechanisms of liver preconditioning. Gastroenterology.

[134] Selzner N., Rudiger H., Graf R., Clavien P. (2003). Protective strategies against ischemic injury of the liver. Gastroenterology.

[135] Alchera E., Dal Ponte C., Imarisio C., Albano E., Carini R. (2010). Molecular mechanisms of liver preconditioning. World J. Gastroenterol..

[136] Richards J.A., Wigmore S.J., Devey L.R. (2010). Heme oxygenase system in hepatic ischemia-reperfusion injury. World J. Gastroenterol..

[137] Wang Y., Shen J., Xiong X., Xu Y., Zhang H., Huang C., Tian Y., Jiao C., Wang X., Li X. (2014). Remote ischemic preconditioning protects against liver ischemia-reperfusion injury via heme oxygenase-1- induced autophagy. PLoS ONE.

[138] Carini R., Alchera E., De Cesaris M.G., Splendore R., Piranda D., Baldanzi G., Albano E. (2006). Purinergic P2Y2 receptors promote hepatocyte resistance to hipoxia. J. Hepatol..

[139] Jin L.M., Jin S.F., Liu Y.X., Zhou L., Xie H.Y., Yan S., Xu X., Zheng S.S. (2012). Ischemic preconditioning enhances hepatocyte proliferation in the early phase after ischemia under hemi-hepatectomy in rats. Hepatobiliary Pancreat. Dis. Int..

[140] Pantazi E., Zaouali M.A., Bejaoui M., Serafin A., Folch-Puy E., Petegnief V., De Vera N., Ben Abdennebi H., Rimola A., Roselló-Catafau J. (2014). Silent information regulator 1 protects the liver against ischemia-reperfusion injury: Implications in steatotic liver ischemic preconditioning. Transpl. Int..

[141] Terada K., Kaziro Y., Satoh T. (2000). Analysis of Ras-dependent signals that prevent caspase-3 activation and apoptosis induced by cytokine deprivation in hematopoietic cells. Biochem. Biophys. Res. Commun..

